# Comprehensive Insights into Tracheoesophageal Fistula Pathophysiology, Diagnosis, Treatment, and Future Directions

**DOI:** 10.1002/advs.202502825

**Published:** 2025-09-02

**Authors:** Gang Li, Zhe Wang, Jia Liu, Shenbo Fu, Longmei Xue, Zijun Liao, Rong Yang, Shanping Huang, Rui Xu, Peng Chen, Yong Chen

**Affiliations:** ^1^ Pediatric Emergency and PICU Northwest Women and Children's Hospital No.1616 Yanxiang Road, Yanta District Xi'an City 710061 China; ^2^ General Practice The Second Affiliated Hospital of Xi'an Jiaotong University Xi'an 710004 China; ^3^ Thoracic Surgery Shaanxi Provincial Cancer Hospital Affiliated to Xi'an Jiaotong University No. 309 Yanta West Road, Xi'an City Xi'an Shaanxi 710061 China; ^4^ Radiotherapy Department Shaanxi Provincial Cancer Hospital Affiliated to Xi'an Jiaotong University No. 309 Yanta West Road, Xi'an City Xi'an Shaanxi 710061 China; ^5^ CT room Shaanxi Provincial Cancer Hospital Affiliated to Xi'an Jiaotong University No. 309 Yanta West Road, Xi'an City Xi'an Shaanxi 710061 China; ^6^ Department of Medical Oncology Shaanxi Provincial Cancer Hospital Affiliated to Xi'an Jiaotong University No. 309 Yanta West Road, Xi'an City Xi'an Shaanxi 710061 China; ^7^ Department of Pathology Shaanxi Provincial Cancer Hospital Affiliated to Xi'an Jiaotong University No. 309 Yanta West Road, Xi'an City Xi'an Shaanxi 710061 China; ^8^ Endoscopy Center Shaanxi Provincial Cancer Hospital Affiliated to Xi'an Jiaotong University No. 309 Yanta West Road, Xi'an City Xi'an Shaanxi 710061 China; ^9^ Neurology Yulin City First Hospital Yulin 719000 China

**Keywords:** congenital TEF, endoscopic therapy, multidisciplinary management, personalized treatment, tracheoesophageal fistula

## Abstract

Tracheoesophageal fistula (TEF) represents a pathological connection between the trachea and esophagus, classified into congenital and acquired categories. Congenital TEF arises from embryological malformations, often coexisting with esophageal atresia (EA), while acquired TEF primarily stems from malignancies, radiotherapy, or trauma. This condition disrupts normal anatomical functions, leading to significant clinical issues, such as aspiration, respiratory infections, and malnutrition. The current review consolidates findings on the etiology, classification, and pathological mechanisms of TEF, emphasizing how genetic, developmental, and external factors shape its occurrence and progression. Diagnostic advancements, including radiological imaging, endoscopic evaluations, and newer monitoring techniques, have refined clinical assessment. Treatment paradigms for TEF vary from minimally invasive endoscopic interventions, to traditional open and innovative surgical approaches. Conservative management, such as nutritional and anti‐infective therapies, remains vital, especially for milder cases. Emerging therapies in regenerative medicine—such as tissue‐engineered scaffolds and platelet‐rich plasmaoffer transformative potential for refractory cases. Future advancements are oriented toward exploring 3D printing, artificial intelligence, and precision medicine. The necessity of multidisciplinary collaboration is underscored in optimizing holistic patient care. While significant progress has improved cure rates and long‐term outcomes, continued research on innovative technologies and clinical trials addressing knowledge gaps in long‐term efficacy remains crucial.

## Graphical abstract

Tracheoesophageal fistula (TEF), a pathological trachea‐esophagus connection, arises congenitally or from malignancy/radiotherapy/trauma, causing aspiration and malnutrition. Diagnosis utilizes imaging and endoscopy. Treatment ranges from endoscopic stenting to surgical repair and conservative management. Regenerative medicine (e.g., scaffolds) and future innovations (AI, 3D printing, precision genomics) offer promise. Multidisciplinary collaboration is crucial for optimizing care and addressing long‐term outcome knowledge gaps.

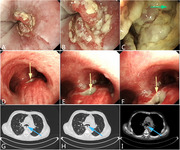

## 1 Introduction

### 1.1 Definition and Pathophysiology Overview of Tracheoesophageal Fistula

**Figure 1:**
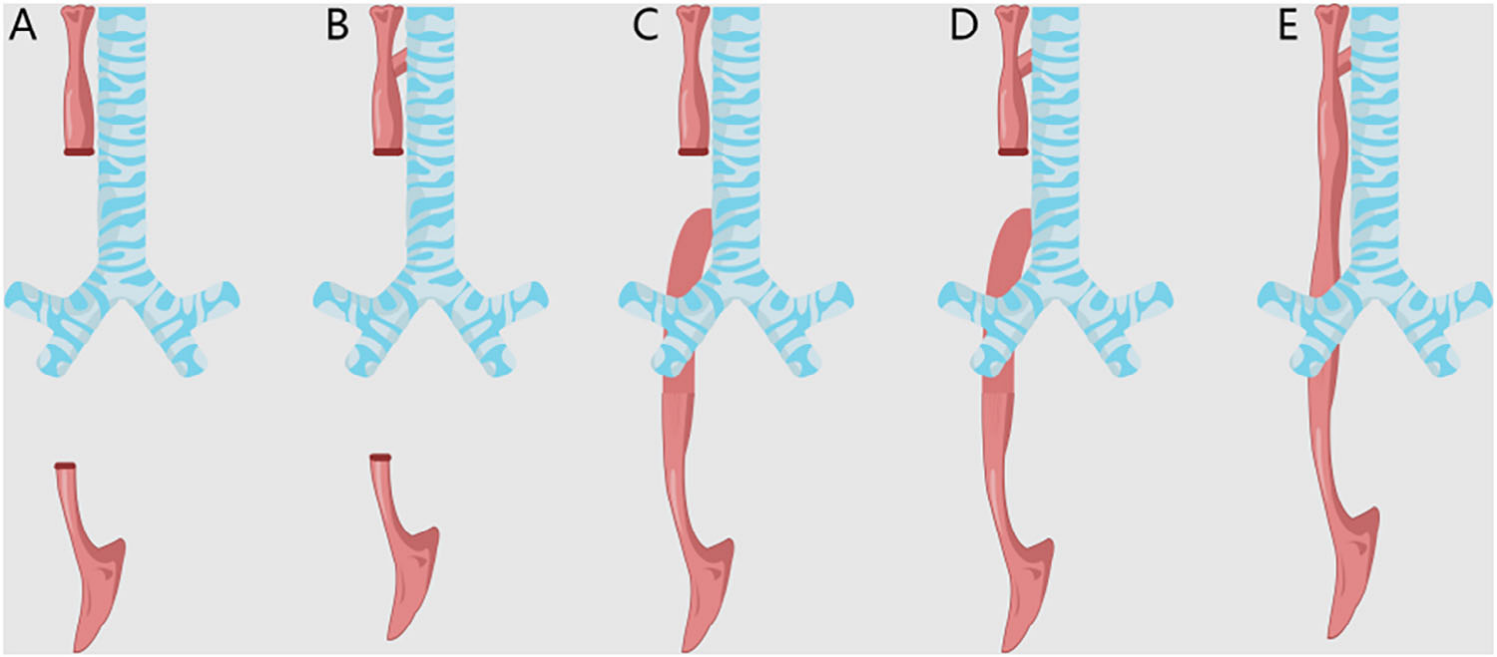
Classification of esophageal atresia and tracheoesophageal fistula. Type A) is EA without an associated TEF, and the trachea is unaffected. Type B) was defined as EA with proximal TEF. Type C) is EA with distal TEF, and type D) is EA with proximal and distal TEFs. Type E) commonly referred to as an H‐type fistula, is a TEF without an associated EA. (EA: esophageal atresia; TEF: tracheoesophageal fistula) (Original work created by the authors).

Tracheoesophageal fistula (TEF) is defined as an abnormal connection between the trachea and esophagus. This pathological condition disrupts the normal anatomical separation and communication between these two structures, leading to significant clinical consequences. TEF is broadly categorized into congenital and acquired forms. Congenital TEF often occurs as part of the spectrum of esophageal atresia (EA) with TEF, with additional rare variants, such as isolated H‐type fistulas, which account for less than 5% of congenital cases (**Figure** **1**). Congenital TEF is are rare developmental abnormality that is typically easily detected during the neonatal period.^[^ ^1^ ^]^ In contrast, acquired TEF arises due to malignancy, trauma, or post‐therapeutic complications, such as intubation or radiotherapy,^[^ ^2^, ^3^ ^]^ and is more common in adults.

Anatomically, the esophagus lies posterior to the trachea and is separated by a robust membranous wall. However, in TEF, a pathological connection is formed, creating a pathway that allows the direct passage of substances between these organs. This abnormality disrupts the protective mechanisms of the airway, predisposing patients to the aspiration of liquids and solids, which can subsequently lead to severe complications such as aspiration pneumonia, chronic respiratory infections, and severe malnutrition. Especially in congenital TEF, associated anomalies such as EA further exacerbate these dysfunctions, resulting in acute respiratory distress and feeding difficulties shortly after birth.^[^ ^2^, ^4^ ^]^

The pathophysiological mechanisms underlying TEF are complex and depend on their congenital or acquired origins. Congenital TEFs likely arise from defective embryogenesis of the foregut, where incomplete septation of the tracheoesophageal ridge during development results in abnormal communication. Acquired TEFs are frequently due to malignancies, particularly esophageal or lung cancer, where progressive tumor invasion erodes the tissue planes to form a fistula. Similarly, iatrogenic and traumatic causes, such as prolonged mechanical ventilation and radiation injury, can lead to ischemia and perforation, precipitating TEF formation.^[^ ^5^, ^6^ ^]^

Clinically, understanding the pathophysiology of TEF is pivotal for guiding treatment strategies. Therapeutic interventions aim to restore anatomical integrity and prevent secondary complications, such as chronic infections and nutritional deficiencies. These principles influence the choice of endoscopic, surgical, or conservative treatment, depending on the severity and etiology of the fistula.^[^ ^2^, ^7^ ^]^

### 1.2 Epidemiology and Disease Burden

The epidemiology of TEF varies significantly depending on its nature. The overall worldwide prevalence of EA, as calculated from national and international databases for congenital anomalies, is estimated to be 2.4 cases (range 1.3–4.6) per 100 000 births.^[^ ^8^, ^9^ ^]^ Congenital TEF is a rare anomaly with an estimated incidence of 1 in 2500 to 1 in 4500 live births. Among these cases, isolated H‐type fistulas are exceedingly uncommon.^[^ ^2^, ^10^ ^]^ Most congenital TEF cases are identified in the neonatal or early pediatric periods, with symptoms such as coughing, choking, cyanosis, and recurrent respiratory infections frequently leading to diagnosis.^[^ ^11^ ^]^

Benign TEFs typically arise in scenarios involving prolonged mechanical ventilation through an endotracheal or tracheostomy tube, excessive cuff pressure, blunt chest or neck trauma, airway injuries, granulomatous mediastinal infections, stent‐induced damage, or the ingestion of foreign objects or corrosive substances.^[^ ^5^, ^12^ ^]^ Granulomatous mediastinal infections, such as tuberculosis, are the leading cause of acquired benign TEFs. However, the widespread use of intubation and tracheostomy has shifted the landscape, with ≈75% of acquired benign TEFs attributable to iatrogenic causes.^[^ ^13^ ^]^

Acquired TEFs are predominantly seen in adults, particularly those with advanced malignancies, such as esophageal or tracheal cancer. The prevalence and incidence data for this type are less comprehensive but suggest that a significant proportion of late‐stage cancer cases experience these complications. Risk factors include the type and location of malignancy, history of radiation therapy, and trauma related to medical interventions.^[^ ^3^ ^]^

The disease burden caused by TEF extends beyond its direct physiological effects on patients. Congenital TEF imposes substantial challenges for families, requiring long‐term specialized care and significant financial and emotional strain. Adults with acquired TEF often face compounded difficulties due to underlying malignancies, resulting in higher hospitalization rates, increased medical costs, and reduced quality of life (QoL).^[^ ^14^ ^]^

Geographically, there appear to be variations in the clinical outcomes of TEF based on access to medical care, availability of diagnostic tools, and expertise in multidisciplinary management strategies. For example, advanced healthcare systems with sophisticated diagnostic imaging and surgical techniques may achieve better early detection and treatment outcomes than regions lacking such resources.^[^ ^10^ ^]^ To treat malignant ERF, timely attention must be paid to correcting tracheal and bronchial contamination and malnutrition.^[^ ^15^ ^]^

### 1.3 Research Background and Objectives of the Review

Over the past few decades, significant progress has been made in understanding the complexities surrounding TEFs. For congenital TEF, advancements in prenatal imaging and surgical techniques, such as thoracoscopic repairs for H‐type fistulas, have improved diagnostic accuracy and treatment outcomes.^[^ ^2^, ^11^ ^]^ The integration of endoscopic stents and novel biomaterials has expanded therapeutic options beyond traditional surgical methods, although their long‐term efficacy and complications remain under investigation.^[^ ^3^ ^]^

However, considerable knowledge gaps persist. For example, the precise mechanisms governing the recurrence and complications of both congenital and acquired TEF remain poorly understood. Additionally, while stents have become a cornerstone in the treatment of acquired TEF, their efficacy varies, and the implementation of newer technologies, such as 3D‐printed stents or tissue‐engineered materials, is still in its infancy.^[^ ^3^, ^14^, ^16^, ^17^ ^]^

The objectives of this review are threefold. First, it aimed to consolidate current knowledge on the etiology, diagnosis, and management of TEF by synthesizing findings from recent studies. Second, it sought to address existing knowledge gaps by comparing different treatment modalities, with an emphasis on both their clinical efficacy and limitations. Finally, this review highlights the critical role of multidisciplinary approaches in optimizing care coordination, emphasizing collaboration among thoracic surgeons, gastroenterologists, radiologists, and other specialists in managing this complex condition.^[^ ^2^, ^14^ ^]^

In summary, this review serves as a comprehensive resource for clinicians and researchers, offering insight into the evolving landscape of TEF diagnosis and treatment while underlining the importance of innovative research and collaborative care pathways.

## 2 Etiology and Pathological Mechanisms and Related Signaling Pathways

### 2.1 Congenital TEF

A congenital TEF arises during embryogenesis due to the incomplete separation of the trachea and esophagus. This developmental anomaly frequently presents as a structural defect that compromises the esophagorespiratory system and causes significant morbidity in early life. Although the exact cause remains unclear, both genetic and environmental factors are believed to contribute.^[^ ^18^ ^]^

#### 2.1.1 Characteristics Related to H‐type Fistula and Esophageal Atresia

The H‐type TEF is a rare congenital variation typically characterized by an isolated fistula without EA. This variant often presents with subtle symptoms such as recurrent aspiration and persistent coughing during feeding, which complicates early diagnosis. Anatomically, H‐type fistulas are described as oblique channels connecting the esophagus and trachea and are often located above the carina, which challenges endoscopic detection in neonates without the adjunct of advanced imaging.^[^ ^18^, ^19^, ^20^ ^]^

In contrast, TEF frequently occurs in conjunction with EA, particularly in commonly encountered Gross Type C variants. In these cases, the upper esophageal segment terminates in a blind pouch, whereas the lower segment fistulates with the trachea. This impedes feeding and ventilation, leading to early respiratory distress and cyanosis. Both conditions necessitate prompt surgical intervention. However, significant challenges arise during repair, including anatomical complexities, vulnerability to tissue damage, and post‐repair complications, such as stricture or recurrence.^[^ ^21^, ^22^ ^]^

#### 2.1.2 Anatomical Abnormalities and Developmental Defects

Congenital TEFs are associated with severe anatomical abnormalities, such as a malformed tracheoesophageal septum and the proximity of the esophageal and airway structures, which can lead to misaligned growth. Notably, malformed complete tracheal rings have been documented in rare cases of congenital TEF, indicating an interplay between localized structural abnormalities and systemic developmental disruptions.^[^ ^7^, ^20^ ^]^

Developmental defects in TEF have also been linked to syndromic genetic associations. Certain patients exhibit concomitant anomalies, such as vertebral, anal, cardiac, tracheoesophageal, renal, and limb syndromes (VACTERL), indicating syndromic involvement in embryonic maldevelopment. Genetic studies emphasize that these abnormalities likely arise from disruptions in signaling pathways such as Sonic Hedgehog (Shh) and related cellular mechanisms responsible for foregut partitioning. However, the underlying mechanisms remain incompletely understood, necessitating further research to identify specific causative factors.^[^ ^18^, ^21^, ^22^ ^]^

### 2.2 Acquired TEF

Acquired TEF represents a pathological communication that develops between the trachea and esophagus after birth, resulting from various extrinsic and intrinsic factors. Unlike congenital TEF, acquired forms manifest across all age groups and are primarily related to disease conditions or medical interventions. Predominantly observed in adults, they can have devastating clinical effects, including life‐threatening infections and respiratory compromise.^[^ ^23^, ^24^ ^]^

#### 2.2.1 Malignant Tumors (e.g., Esophageal Cancer, Lung Cancer) and Treatment‐Related Mechanisms

Malignancy, particularly due to esophageal and lung carcinomas, underlies the development of a significant proportion of acquired TEFs.^[^ ^25^ ^]^ These tumors may erode directly into the adjacent tissues because of their aggressive invasive nature, leading to fistulization. For instance, esophageal cancer with transmural invasion or metastatic lymph node involvement can breach the integrity of the esophageal and tracheal walls and establish a pathological connection.^[^ ^23^, ^26^ ^]^

**Figure 2:**
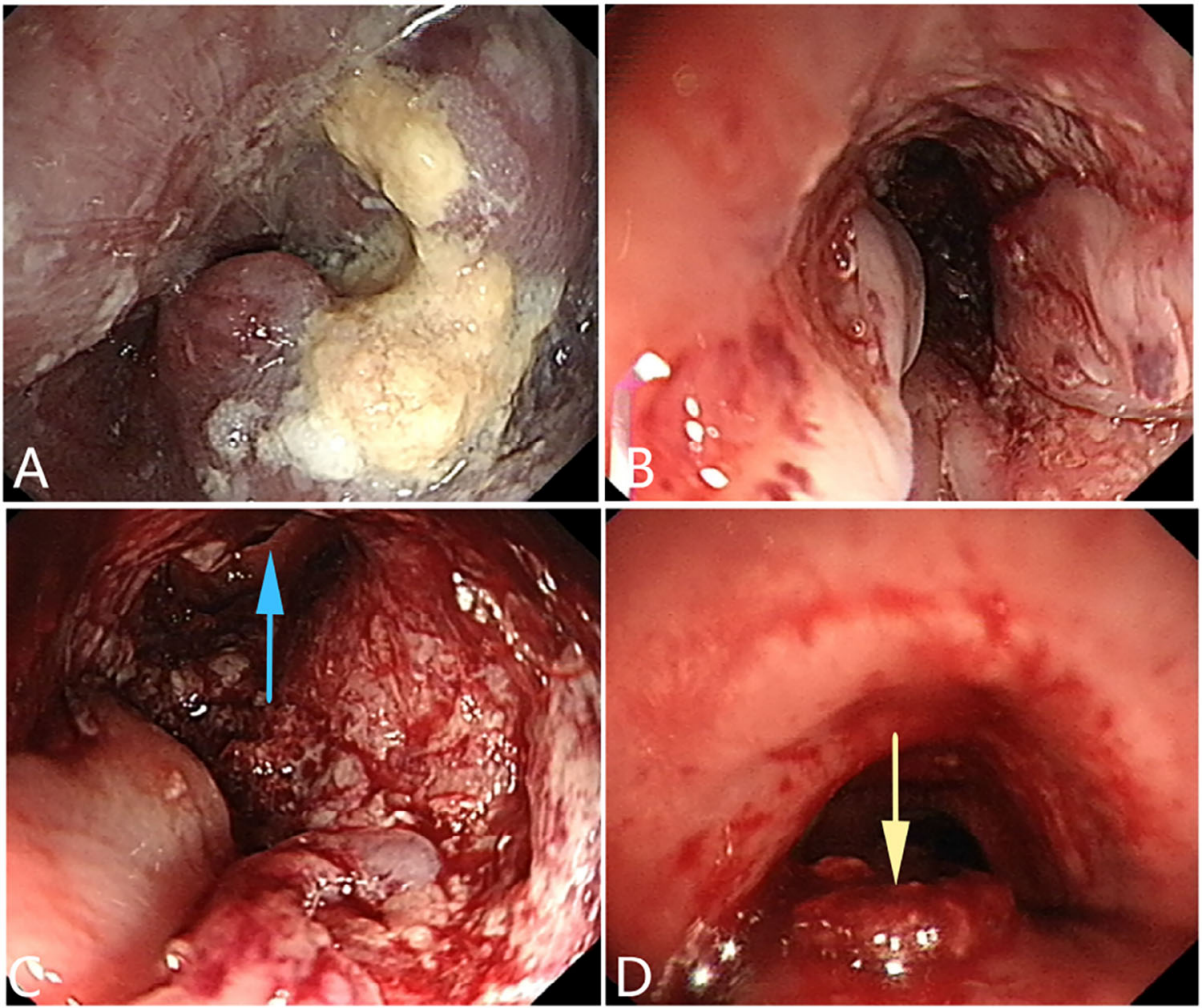
An esophageal malignant tumor is invading the trachea, leading to a tracheoesophageal fistula. A, B) Gastroscopy showing a malignant tumor in the esophageal cavity, leading to esophageal stenosis. C) Gastroscopic image showing the formation of a fistula in the middle of the lesion, with the blue arrow indicating the fistula opening. D) Bronchoscopy showing that the tumor has invaded the tracheal membrane, and a fistula can be seen at the center of the lesion. The yellow arrow indicates the fistula opening. (Original work created by the authors).

**Figure 3:**
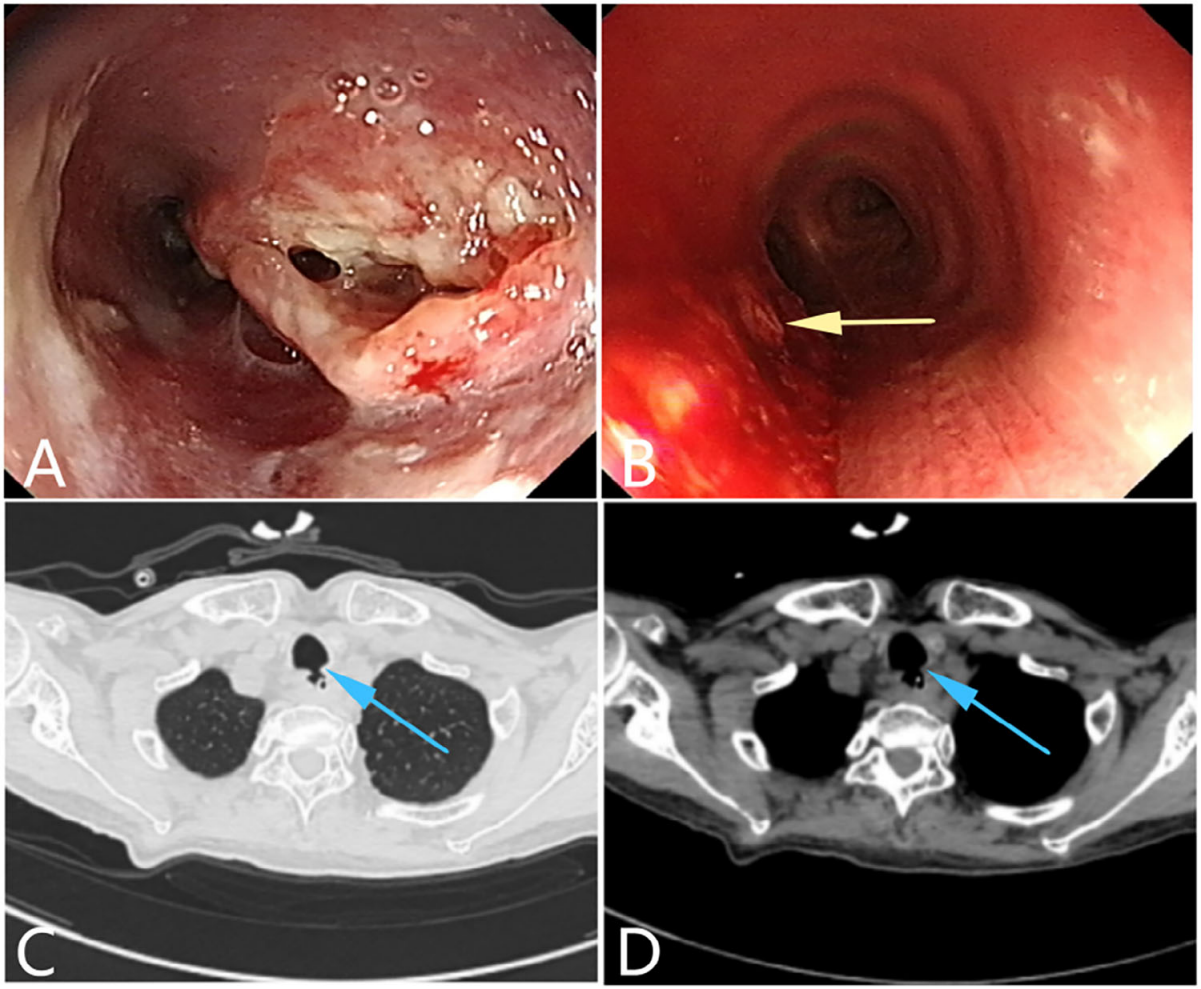
An esophageal malignant tumor invades the trachea, leading to a tracheoesophageal fistula. A) Gastroscopy revealed an ulcerative malignant tumor in the esophageal cavity. B) Bronchoscopy showing the formation of a fistula on the left wall of the trachea with necrotic material and blood flowing out from it. C, D) CT scan showing the pathway between the esophagus and the trachea. (Original work created by the authors).

**Figure 4:**
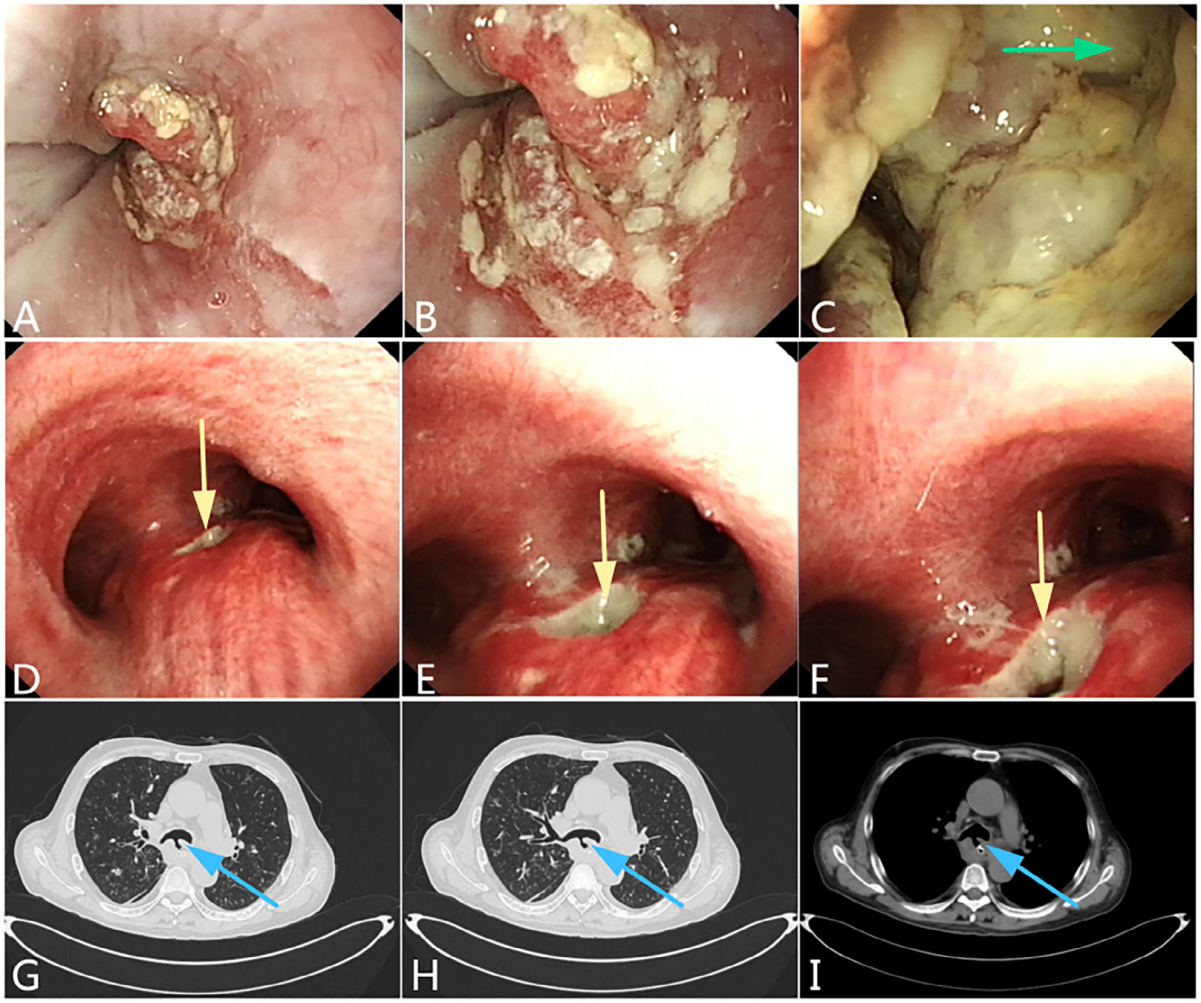
An esophageal malignant tumor is invading the trachea, leading to a tracheoesophageal fistula. A–C) Gastroscopy showing malignant tumors of the esophagus causing narrowing of the esophageal cavity. A fistula can be observed in the middle of the lesion, and an extensive white coating is attached around it. The green arrow indicates the fistula. D–F) Bronchoscopy showing the formation of a fistula in the tracheal membrane with an extensive white coating attached around it. G–I) CT scan showing the formation of a pathway between the esophagus and trachea. (Original work created by the authors).

In a large case series, most malignant TEFs were attributed to esophageal cancer, accounting for 92% of all TEF cases. Lung cancer was identified as the cause in 7% of cases, whereas mediastinal tumors contributed to 1% of cases^[^ ^15^, ^27^ ^]^ (**Figures** **2**, **3**, **4**).

**Figure 5:**
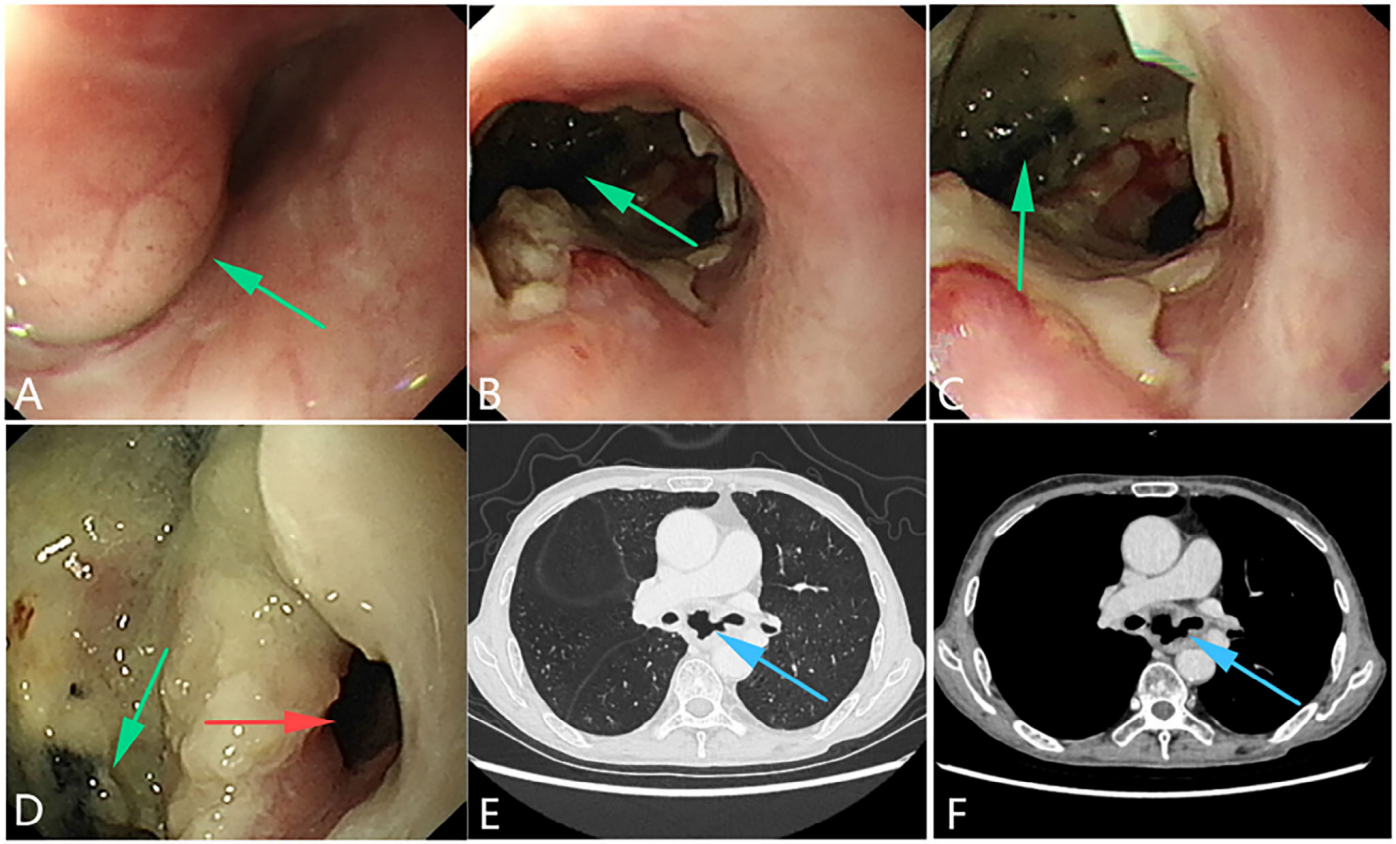
Tracheoesophageal fistula formed after radiation therapy for malignant lung tumors. A) Gastroscopy showed that a malignant lung tumor was compressing the esophageal cavity, causing it to narrow; the surface mucosa was intact and had not yet been broken through. B–D) The patient underwent radiation therapy, and a follow‐up gastroscopy 2.5 months later revealed an esophageal fistula with an extensive white coating attached to the surface. The green arrow represents the fistula, and the red arrow represents the esophageal cavity. E, F) A CT scan can reveal the pathway between the esophagus and trachea. (Original work created by the authors).

Radiotherapy and chemotherapy, while pivotal in malignancy management, contribute to TEF development by delaying adverse effects, including tissue necrosis and scarring. Prolonged radiation exposure can induce ischemic injury, impair local tissue integrity, and predispose patients to fistula formation. Therapeutic interventions and disease burden synergistically increase risk^[^ ^28^, ^29^ ^]^(**Figure** **5**).

**Figure 6:**
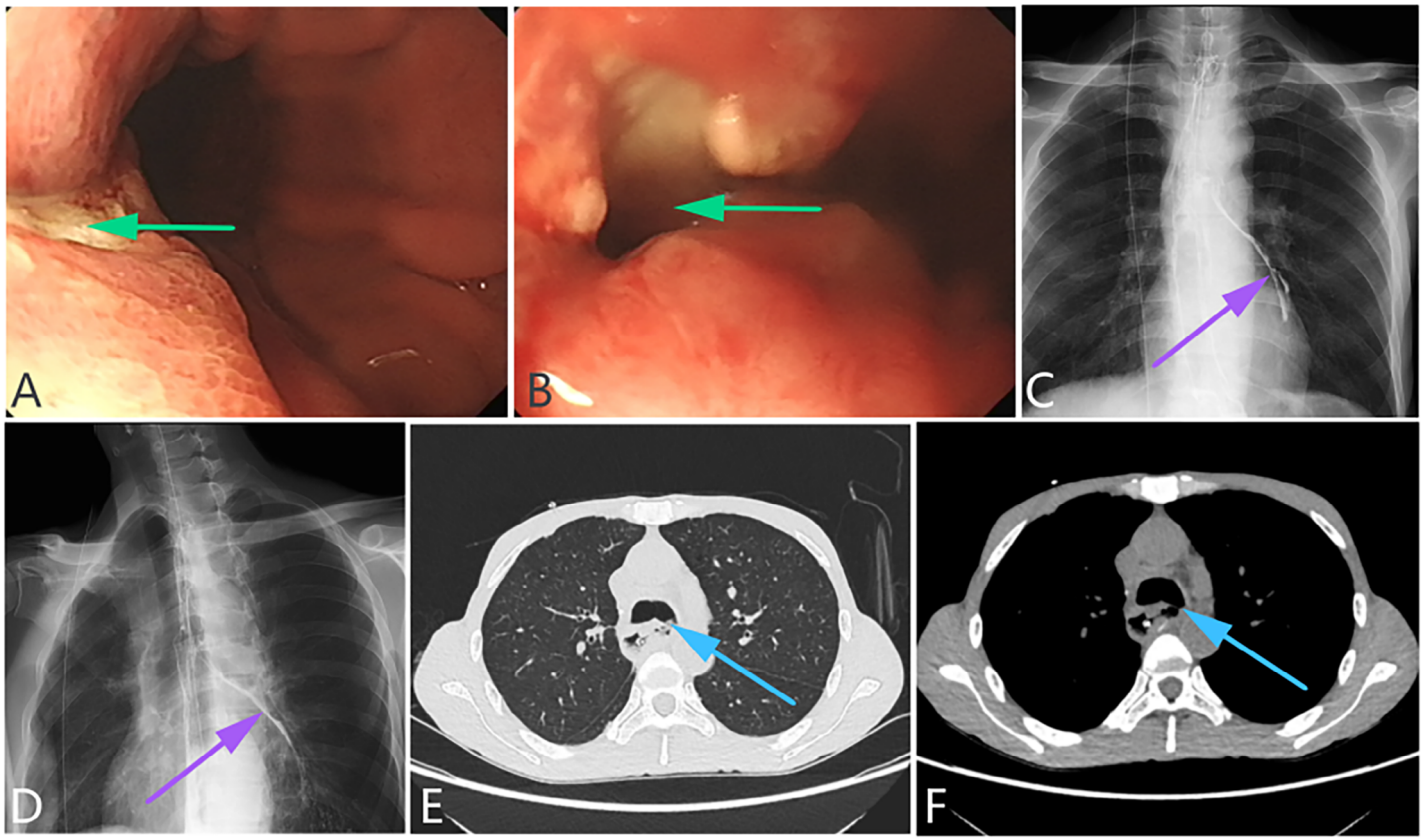
Postoperative anastomotic recurrence of esophageal malignant tumors leads to fistula formation. A, B) After surgery for malignant esophageal tumors, the anastomotic site recurred, and a fistula formed locally. C, D) Upper gastrointestinal image showing a fistula and left bronchial image. E, F) CT scan showing a pathway between the anastomotic site and the left main bronchus. (Original work created by the authors).

**Figure 7:**
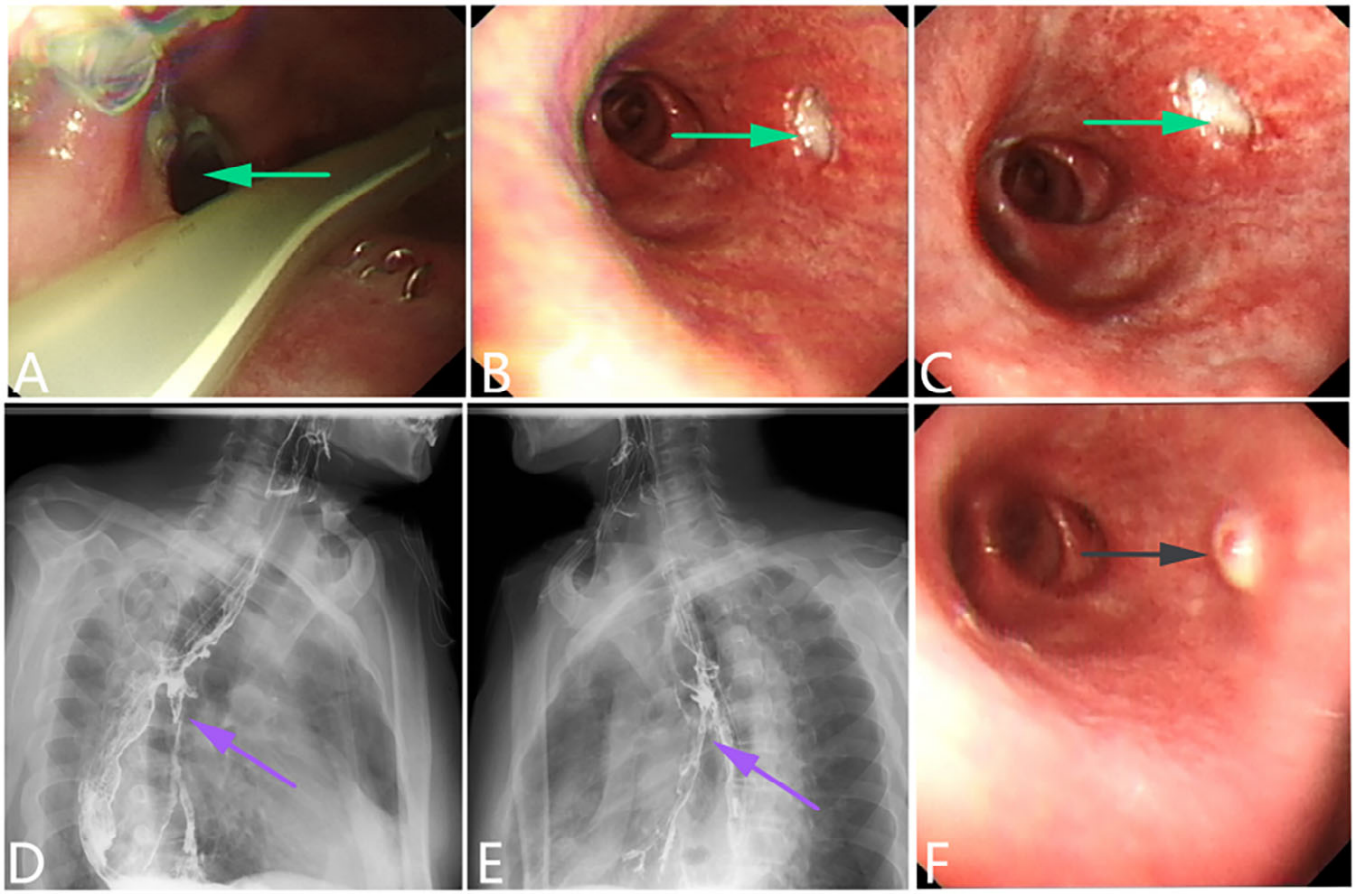
Postoperative anastomotic fistula in esophageal malignant tumor. A) Gastroscopy revealed a fistula at the anastomotic site, with some residual anastomotic nails visible, and an indwelling gastric tube. B, C) Under bronchoscopy, the middle right bronchial fistula can be seen, with a white coating attached to the inside of the fistula. D, E) Upper astrointestinal image showing a tracheoesophageal fistula with imaging of the right bronchus. F) After conservative treatment for 48 days, the patient underwent a follow‐up bronchoscopy, which showed that the original right middle bronchial fistula had healed, and granulation tissue proliferation could be seen on the surface. (Original work created by the authors).

Comprehensive surgery is the primary treatment option for esophageal cancer. In such procedures, after preparing the tubular stomach, the esophagus is anastomosed to it. However, a postoperative cervical anastomotic fistula occurs in ≈16.6% of cases^[^ ^30^ ^]^ and is one of the most common complications of esophagectomy in cancer treatment^[^ ^31^ ^]^ (**Figures** **6** and **7**).

#### 2.2.2 Traumatic Causes (Tracheal Intubation and Radiotherapy)

Trauma, particularly prolonged or improper tracheal intubation, is a major contributor to TEF development. Endotracheal tubes exert direct pressure on the tracheal wall, potentiating ischemia and necrosis. Similarly, patients receiving nasogastric feeding for extended periods are at risk because tube placement can compromise esophageal blood flow and promote inflammation at pressure points, ultimately leading to fistula establishment.^[^ ^24^, ^26^ ^]^ Damage from foreign objects is also a contributing factor, such as the detachment of removable dentures in elderly individuals, which can lead to the formation of a TEF.^[^ ^32^ ^]^

**Figure 8:**
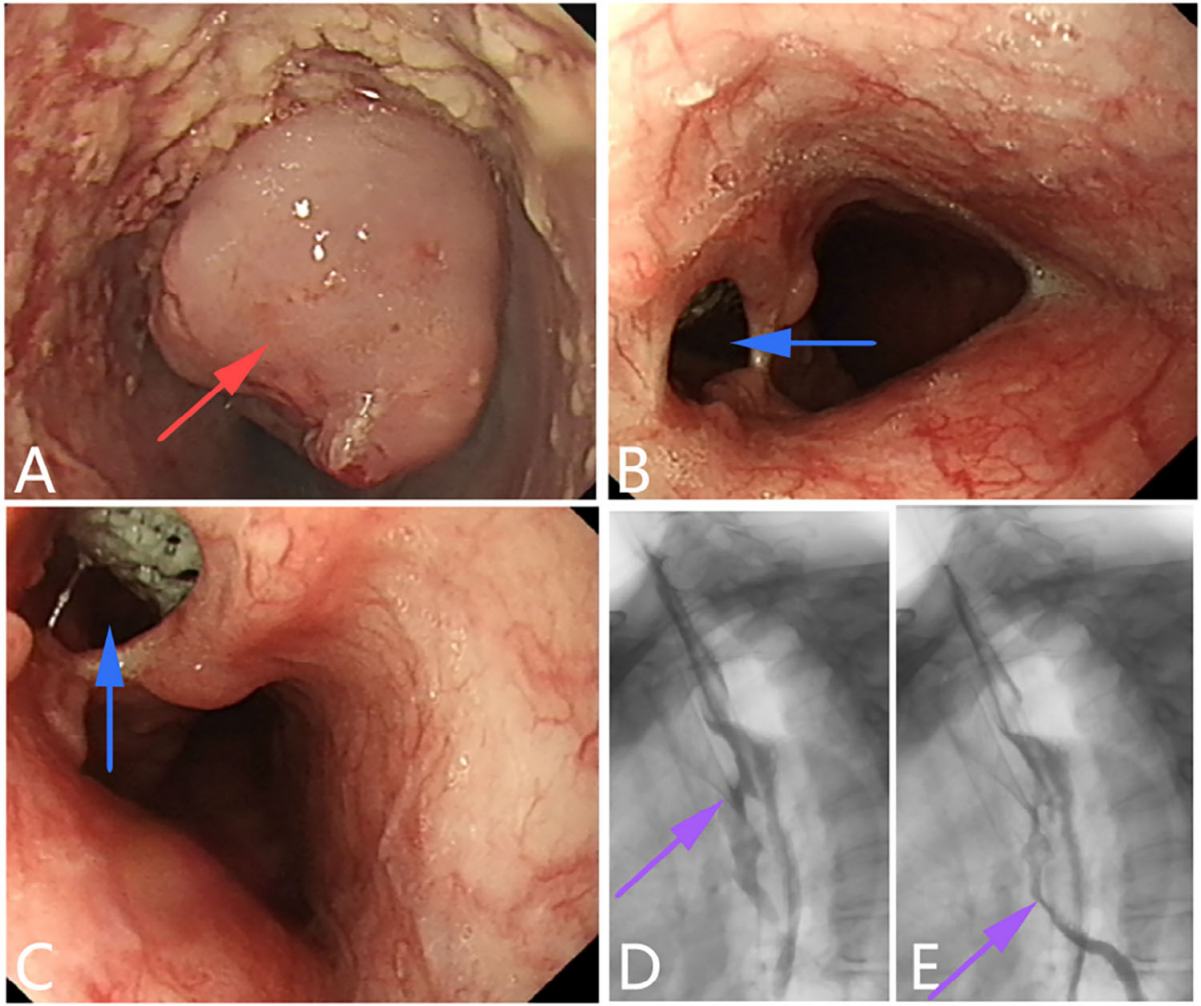
A tracheoesophageal fistula formed after radiotherapy and chemotherapy for a malignant esophageal tumor. A) Gastroscopy revealed a malignant tumor of the esophagus with narrowing of the esophageal lumen. The surrounding mucosa was rough and covered with a large white coating. B, C) After radiotherapy and chemotherapy, the original mass disappeared, and a fistula formed locally. Retention of the esophageal residue is observed in the fistula. D, E) Upper gastrointestinal image showing a tracheoesophageal fistula and tracheal and bronchial images. (Original work created by the authors).

**Figure 9:**
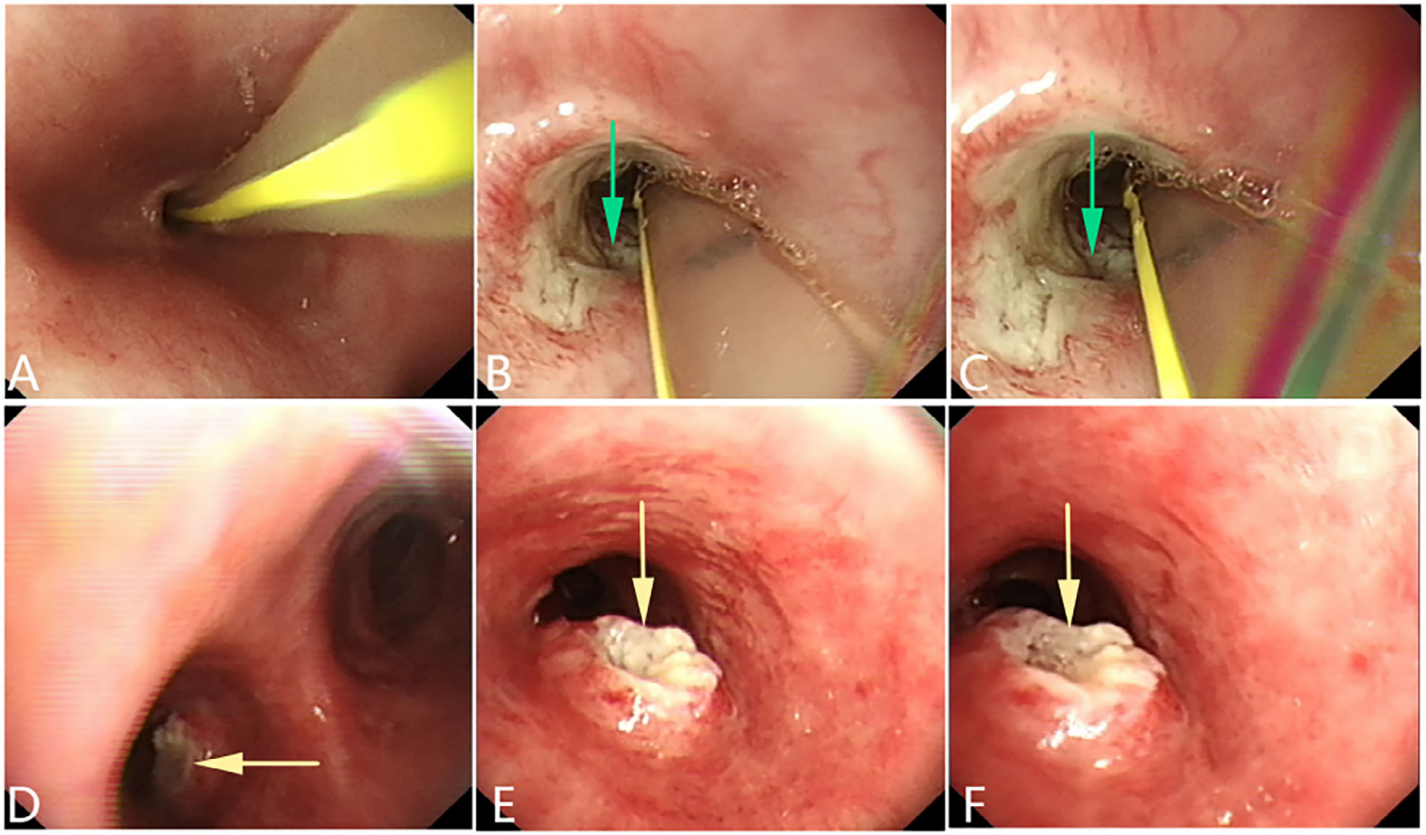
Left main esophageal fistula forming after radiotherapy for a malignant esophageal tumor. A) Gastroscopy detected a malignant esophageal tumor. After radiotherapy, the esophageal lumen narrowed, necessitating the insertion of a gastric tube. B, C) Surface erosion of malignant esophageal tumor lesions with an extensive white coating and visible fistula formation in the middle area are seen. D–F) Under bronchoscopy, a malignant tumor can be seen invading the left main bronchus, and fistula formation can be observed in the central area. (Original work created by the authors).

**Figure 10:**
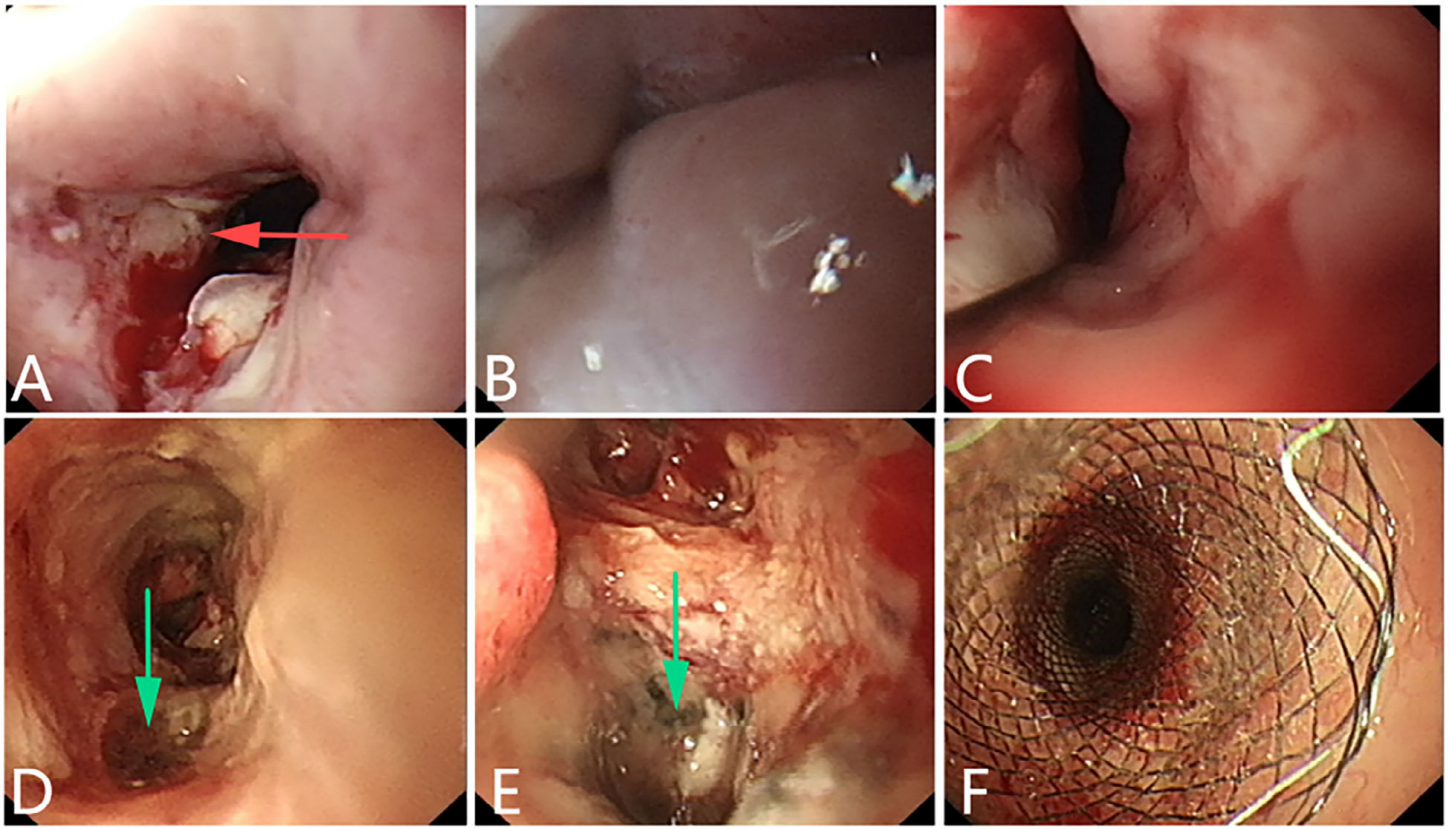
A tracheoesophageal fistula developed after radiotherapy for a malignant esophageal tumor, necessitating the placement of a covered metal stent for treatment. A–C) After receiving radiotherapy, malignant tumors of the esophagus can be seen with tissue edema and obvious erosion on the surface, leading to esophageal stenosis. D, E) A large fistula can be observed in the middle of the lesion with an extensive white coating adhering to the surface. F) Implantation of a covered metal stent in the esophagus is noted. (Original work created by the authors).

Radiotherapy further exacerbates the risk of TEF in predisposed patients, especially those undergoing treatment for mediastinal malignancies. Tissue necrosis induced by high‐dose radiation is a significant concern in the mediastinum, where the esophageal and tracheal structures are particularly vulnerable. Case studies reveal that radiation‐induced TEFs often occur months after treatment, emphasizing the necessity for monitoring long‐term post‐therapeutic effects^[^ ^26^, ^33^, ^34^ ^]^ (**Figures** **8**, **9**, **10**).

#### 2.2.3 Role of Infection and Immune Response

Understanding the role of infection in the formation of acquired TEF is of paramount importance. Chronic infections, such as pulmonary tuberculosis, can compromise tracheal and esophageal tissue integrity, leading to microperforations that coalesce into full fistulas. Superimposed bacterial proliferation exacerbates local tissue destruction and creates conditions conducive to fistula development. In some cases, fistulization occurs because of systemic inflammatory responses involving autoimmune dysfunction or immunocompromised states.^[^ ^34^, ^35^, ^36^ ^]^

From an immunopathological standpoint, heightened inflammatory responses amplify tissue damage either through direct immune‐mediated cytotoxicity or by perpetuating necrotizing inflammation. Studies have indicated that significant cytokine dysregulation and immune activation may predispose tissues to structural breakdown, highlighting the complex interplay between infection, immunity, and TEF progression.^[^ ^37^, ^38^ ^]^

Despite a growing body of evidence, significant gaps remain in our understanding of the etiological and pathological nuances of both congenital and acquired TEFs. The multifactorial nature of these abnormalities necessitates continued research to unravel their underlying mechanisms and optimize effective prevention and treatment strategies.

### 2.3 TEF Signal Pathways and Targeted Therapy Applications

The pathogenesis of EA/TEF involves multiple signaling pathways. A comprehensive understanding of these pathways is essential for developing targeted therapies.

#### 2.3.1 Key Signal Pathways in EA/TEF

##### Genetic heterogeneity of EA/TEF

The etiology of EA/TEF is complex and involves the interplay of various factors, including genetic, environmental, and epigenetic influences.^[^ ^39^ ^]^ Although research over the past few decades has identified several genes and signaling pathways associated with EA/TEF, the precise genetic underpinnings remain largely unknown.^[^ ^40^, ^41^ ^]^

Recent advancements in genomic technology have offered new insights into the genetic heterogeneity of EA/TEFs. A retrospective study^[^ ^40^ ^]^ analyzed genetic testing data from 212 patients with EA/TEF admitted to a level four children's hospital between 2011 and 2023. The findings revealed that these patients could be categorized into three groups: complex/syndromic EA/TEF (accompanied by other significant anatomical abnormalities), isolated/nonsyndromic EA/TEF, and isolated EA. The results indicated that children with complex/syndromic EA/TEF exhibited a higher rate of genetic testing, particularly karyotype analysis and exome sequencing, with positivity rates of 100% and 21.6%, respectively. In contrast, the positivity rate of genetic testing for isolated/nonsyndromic EA/TEF was lower, implying that its genetic mechanisms may differ from those of the complex type. These findings underscore the considerable genetic heterogeneity in EA/TEF and highlight the significance of genomic testing, such as exome or whole‐genome sequencing, for elucidating its genetic basis.

##### Key genes and signaling pathways involved in the process of foregut separation

The esophagus and trachea arise from a shared foregut tube, and their separation involves a series of precisely coordinated cellular and molecular processes.^[^ ^42^ ^]^ Transcription factors, such as SOX2, NKX2.1, and GATA4, are critical for foregut and esophagus development.^[^ ^42^ ^]^ Research indicates that Abnormal SOX2 expression may impede foregut separation, resulting in EA and TEFs (EA/TEF).^[^ ^43^ ^]^ Furthermore, dysregulation of NKX2.1 is also linked to the onset of EA/TEF, underscoring its vital role in esophageal and tracheal differentiation.^[^ ^43^ ^]^

Investigation of animal models and induced pluripotent stem cells (iPSCs) has illuminated the signaling pathways pertinent to esophageal development, notably the BMP, Wnt, and Notch pathways.^[^ ^39^, ^42^ ^]^ For instance, the BMP signaling pathway is crucial for foregut separation and morphogenesis in the esophageal epithelium.^[^ ^44^ ^]^ Additionally, the transcription factors ZFHX3, TRPS1, and CHD7 have been linked to the onset of EA/TEF.^[^ ^41^ ^]^ These genes are expressed during embryonic development, and their mutations may disrupt normal separation of the foregut.^[^ ^41^ ^]^

Epigenetic regulation plays a crucial role in EA/TEF development. A study conducted on EA twins identified significant hypermethylation and hypomethylation within their genomes affecting multiple key genes, including those in the Rho GTPase pathway.^[^ ^39^ ^]^ These epigenetic alterations may disrupt gene expression, thereby hindering foregut separation and esophageal development. Furthermore, the implementation of tissue‐specific molecular detection and RNA sequencing technologies offers innovative tools for elucidating the epigenetic mechanisms underlying EA/TEF.^[^ ^40^ ^]^

The histological characteristics of EA/TEF indicate that its etiology may not be linked to cell fate determination but rather to abnormalities in tissue structure and signaling pathways. Significant differences in gene expression and tissue architecture exist between TEF tissue and the normal esophagus; notably, the overexpression of genes is associated with smooth muscle contraction and downregulation of BMP signaling pathways. These findings imply that TEFs represent a distinct tissue type linked to myofibroblast activation and fibrotic processes.^[^ ^44^ ^]^

#### 2.3.2 Challenges and Future Directions

Although some studies have identified genes and signaling pathways associated with EA/TEF, the genetic and molecular mechanisms underlying these conditions require further investigation.^[^ ^40^, ^41^ ^]^ Future research should integrate genomics, epigenetics, and single‐cell sequencing techniques to comprehensively analyze the etiology of EA/TEF.^[^ ^39^, ^40^ ^]^ Additionally, developing disease and animal models based on iPSCs will aid in elucidating the molecular mechanisms underlying foregut separation and esophageal development, thereby offering a theoretical basis for personalized treatment of EA/TEF.^[^ ^39^, ^42^, ^43^ ^]^

Although these signaling pathways present promising therapeutic targets, their clinical translation is challenged by issues such as targeting specificity, potential side effects, and intricate interactions among various pathways. Future research should prioritize combinatorial targeting strategies and personalized approaches tailored to the distinct pathophysiology of fistulas in individual patients.

A comprehensive understanding of ETF‐related signaling pathways and their therapeutic potential lays the groundwork for developing innovative treatment strategies that can significantly enhance the outcomes of patients facing this complex condition.

## 3 Clinical Presentation and Diagnostic Methods

### 3.1 Clinical Presentation

#### 3.1.1 Typical Symptoms and Classification: Cough, Cyanosis, and Malnutrition

TEFs present with a constellation of symptoms, primarily driven by abnormal communication between the trachea and esophagus, leading to the disruption of normal physiological processes.

Cough is a hallmark symptom of TEF and often has a paroxysmal quality and is closely linked to feeding, as liquids or saliva entering the esophagus frequently escape into the trachea via the fistula. In more severe cases, or when left untreated, the cough becomes persistent, exacerbating respiratory distress. The cough is further aggravated by aspiration, which fuels recurrent pneumonia, which is a common complication in patients with TEFs.^[^ ^6^, ^25^, ^45^ ^]^

Cyanosis results from hypoxia and is linked to intermittent airway obstruction and aspiration, particularly during feeding. This manifestation is more pronounced in infants, where immature respiratory mechanics exacerbate the condition. Cyanotic episodes are an immediate clinical concern and often require urgent intervention to prevent further complications.^[^ ^38^ ^]^

Malnutrition results from the dual effects of feeding difficulties and chronic aspiration. Recurrent nutritional loss, paired with vomiting or regurgitation, results in failure to thrive in neonates and infants. A study on post‐thoracoscopic repair of TEF in children showed that 31.5% of the participants experienced significant body mass deficiency, reflecting the nutritional consequences of this condition. Chronic malnutrition further results in stunted growth, as evidenced by 28.6% of children in the same cohort presenting with both low body mass and reduced stature.^[^ ^29^ ^]^

A classification system for TEF symptoms would be beneficial for correlating symptom severity with anatomical anomalies. For instance, more severe symptoms, such as significant cyanosis or frequent aspiration pneumonia, are often indicative of larger fistulas or difficult‐to‐access anatomical configurations.^[^ ^38^ ^]^ Correlating symptoms with the anatomical type of TEF, as seen in the Gross and Vogt classifications, further guides therapeutic decision‐making.

#### 3.1.2 Symptom Pattern Differences Between Children and Adults

The clinical presentation of TEF exhibits notable differences between pediatric and adult populations, which are driven by developmental and pathophysiological factors.

Acute versus chronic presentation is a defining feature in different age groups. In infants, severe symptoms, such as choking, aspiration, and cyanosis, often manifest immediately after birth or during initial feeding attempts. Symptoms such as frequent coughing, respiratory distress, and poor feeding efficiency dominate the clinical picture of congenital TEF in neonates. Conversely, adult patients with undiagnosed congenital H‐type TEF present more insidiously and experience chronic respiratory symptoms, such as recurrent pneumonia and persistent coughing, often exacerbated by swallowing difficulties.^[^ ^38^, ^46^ ^]^ Misdiagnosis or delayed diagnosis is commonly observed in adults due to these subtle chronic symptoms.

Age‐specific symptoms reflect physiological and developmental differences between age groups. In neonates, inefficient feeding leads to short‐term aspiration and long‐term nutritional deficits, whereas older adults primarily experience complications related to chronic reflux or infection. Multichannel intraluminal impedance with pH monitoring (MII/pH) has demonstrated that gastroesophageal reflux disease (GERD) is common in children after TEF repair. In a study of children with repaired TEF, 10 of 19 patients were diagnosed with GERD, including acid and non‐acid reflux, further contributing to the associated symptoms and complications.^[^ ^29^ ^]^ Adults, particularly those with congenital TEF present in later life, show fewer symptoms of malnutrition but often bear a heavier burden of respiratory sequelae, such as tracheomalacia or lung infections.^[^ ^46^ ^]^

Psychosocial impact is another consideration that differs widely between children and adults. Feeding difficulties and chronic symptoms experienced from birth lead to a cascade of psychological stresses in pediatric patients and their families. In adults, chronic and sometimes misdiagnosed TEF may result in reduced QoL due to persistent respiratory discomfort and challenges, such as social embarrassment over symptoms, including chronic coughing during meals. Therefore, early detection and personalized management tailored to a patient's age and symptomatology are critical for improving outcomes across all age groups.

### 3.2 Radiological Diagnosis

#### 3.2.1 Application and Limitations of X‐ray and CT Scan

Radiological imaging serves as the cornerstone for the diagnosis of TEF, offering critical insights into the anatomical and physiological disruptions caused by fistulas.

Radiography is often the first‐line imaging modality for neonates with suspected TEF. In cases involving EA, radiography reveals air‐filled esophageal pouches and complete disconnection of the esophagus and stomach due to blockage. However, the lack of direct visualization of the fistulous tract presents a limitation, particularly for H‐type fistulas.^[^ ^38^ ^]^ Contrast radiography may further highlight the fistula route; however, misinterpretation and operator errors often lead to false negatives, contributing to diagnostic delays.

CT provides a more detailed and structured visualization, particularly in complex or atypical cases. High‐resolution CT imaging with contrast enhancement can delineate the connection between the esophagus and trachea, aiding in precise diagnosis. However, pediatric usage may be limited by radiation concerns, prompting clinicians to reserve this modality for cases in which other techniques fail to confirm the diagnosis. CT scans are generally more applicable in adult populations, where chronic symptoms necessitate thorough anatomical mapping before surgical repair.^[^ ^47^ ^]^

In comparing the two, CT scans are superior in detecting subtle and atypical fistulas but may not be warranted as a routine diagnostic approach due to cost and resource constraints.

#### 3.2.2 Esophagography and Related Imaging Techniques

**Figure 11:**
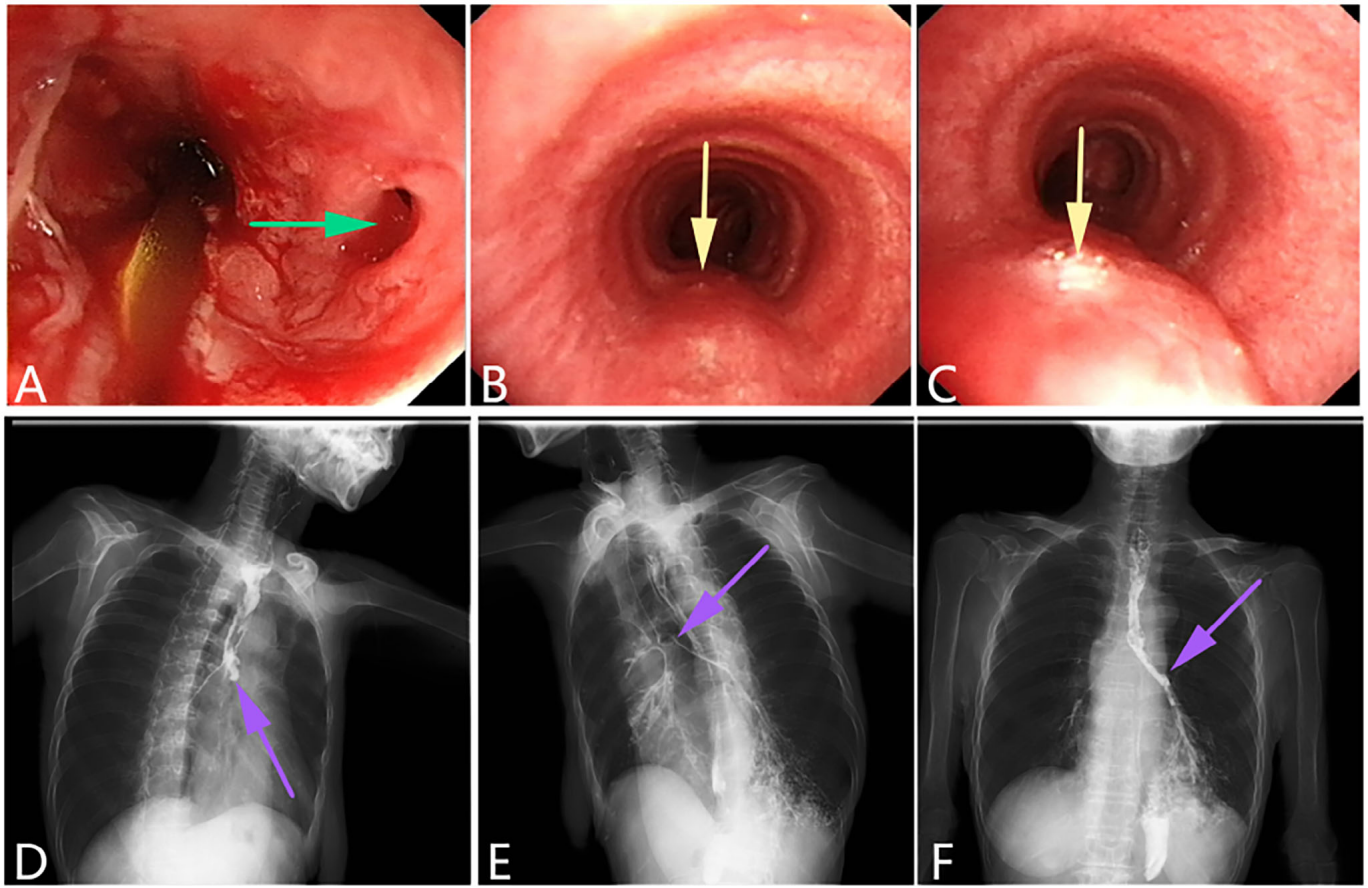
Postoperative anastomotic fistula in esophageal malignant tumors. A) Visible anastomotic fistulas on gastroscopy. B, C) Bronchoscopy showing a fistula on the tracheal membrane. D–F) Upper gastrointestinal image showing a tracheoesophageal fistula and bilateral bronchial images. (Original work created by the authors).

Esophagography remains a key imaging option when initial modalities, such as radiography, are inconclusive. This technique involves contrast fluoroscopy to visualize abnormal communication between the esophagus and trachea. Esophagography is often critical for the diagnosis of H‐type fistulas. By modifying patient positioning or utilizing adjunct techniques, such as guidewire placement, diagnostic sensitivity improves significantly^[^ ^48^ ^]^ (**Figure** **11**).

Other imaging modalities, such as magnetic resonance imaging (MRI), are less commonly used due to lower specificity in identifying small fistulas. However, they may provide valuable adjunctive information in complex cases with extensive tissue involvement or recurrent fistula formation.

### 3.3 Endoscopic Examination and Additional Methods

#### 3.3.1 Complementary Roles of Bronchoscopy and Esophagoscopy

Bronchoscopy plays an instrumental role not only in confirming TEF but also in evaluating the trachea for associated anomalies or damage. Flexible bronchoscopy provides direct visualization of the fistula opening and is invaluable for determining its precise location relative to adjacent anatomical landmarks. Furthermore, bronchoscopic findings are pivotal in planning surgical and endoscopic management.^[^ ^46^, ^49^ ^]^

Conversely, esophagoscopy focuses more on the esophageal part of the anomaly. Direct inspection allows visualization of the fistulous opening within the esophagus, while also enabling therapeutic interventions, such as stent placement in select cases.^[^ ^50^ ^]^

The combined use of these modalities strengthens diagnostic accuracy, with bronchoscopy assessing the tracheal side and esophagoscopy assessing the esophageal side of the fistula.

#### 3.3.2 Endoscopic Biopsy and Novel Biomarker Detection

Endoscopic biopsy, although less utilized in exclusively diagnosing TEF, is relevant in distinguishing TEF from other conditions. Biopsies may also reveal evidence of secondary complications, such as esophagitis or metaplasia, guiding postoperative therapy. Novel biomarker detection remains in the nascent stages but holds potential for the noninvasive categorization of fistula etiology.^[^ ^51^ ^]^

#### 3.3.3 Applications of Multichannel Intraluminal Impedance and pH Monitoring

The combination of multichannel intraluminal impedance and pH monitoring (MII/pH) has revolutionized the diagnostic landscape for reflux‐associated complications in patients with TEFs. MII/pH examines both acid and non‐acid reflux, allowing for a detailed assessment of esophageal exposure to the gastric contents.^[^ ^52^, ^53^, ^54^, ^55^ ^]^

A clinical study involving 57 children with congenital EA evaluated gastroesophageal reflux using pH and impedance monitoring, and retrograde bolus movements were detected in 52.6% of the children detected retrograde bolus movements (RBM).^[^ ^56^ ^]^ By correlating reflux indices with symptomatology, clinicians can optimize management strategies and significantly improve patient outcomes.

Thus, a thorough diagnosis that integrates clinical manifestations with radiological methods and endoscopic examination ensures a comprehensive evaluation and effective management of TEF across diverse patient populations.

## 4 Treatment Methods Overview

### 4.1 Endoscopic Treatment

Endoscopic treatment plays a pivotal role in the management of TEF and offers minimally invasive options for select cases. Techniques employed in endoscopic treatment target both temporary and permanent closure of the fistula while mitigating complications such as infection and recurrent fistula. This section discusses the placement of covered metallic stents and other closure techniques, such as chemical electrocoagulation and endoscopic submucosal dissection, combined with double‐action tissue clamp closure of the TEF.

#### 4.1.1 Placement and Optimization of Covered Metallic Stents

**Figure 12:**
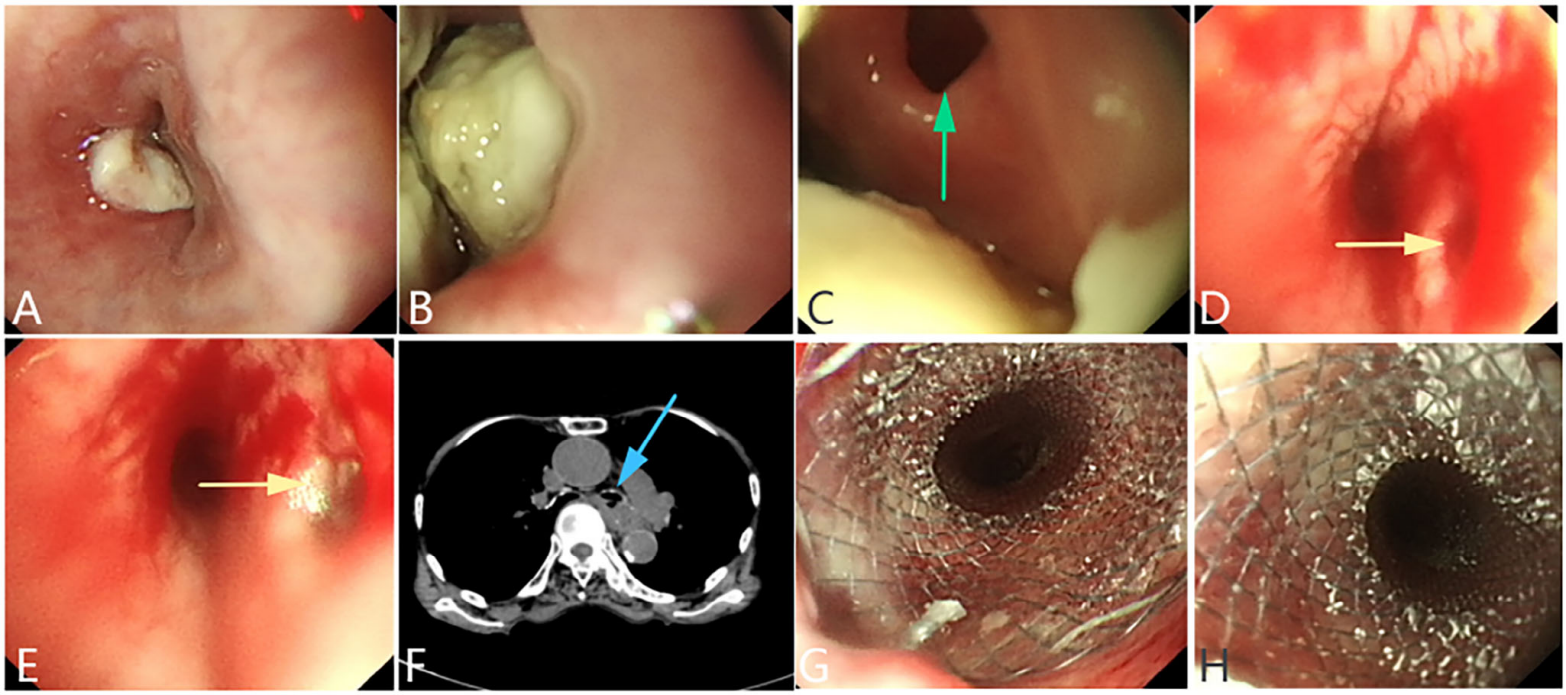
A malignant esophageal tumor invading the bronchus, leading to a left main bronchial fistula in the esophagus, and a covered metal stent was placed on the esophageal side for treatment. A, B) A malignant esophageal tumor causes narrowing of the esophageal lumen, with an extensive white coating on the surface of the lesion. C) A fistula is observed in the middle of the lesion. D, E) Bronchoscopy showing the left main bronchial fistula opening. F) CT image showing the left main bronchial fistula of the esophagus. G, H) displays the esophageal side covered with a metallic stent. (Original work created by the authors).

The placement of covered metallic stents has become a critical option in TEF management, particularly in malignant or postoperative cases. These stents are designed to create a barrier that prevents communication between the trachea and esophagus, thereby alleviating symptoms such as aspiration and improving the QoL. Self‐expandable stents, commonly made of materials such as nitinol or stainless steel, are favored because of their flexibility and durability^[^ ^28^, ^57^, ^58^ ^]^(**Figure** **12**).

The procedure for stent placement involves patient‐specific preprocedural assessments, including imaging and endoscopic visualization, to determine the fistula size and location. Placement is typically performed under general anesthesia with fluoroscopic or endoscopic guidance to ensure accurate positioning. Despite its efficacy, complications, such as stent migration, local infection, obstruction, and fistula recurrence, remain challenging. Long‐term complications, including pressure necrosis and granulation tissue formation at stent edges, require periodic monitoring.^[^ ^28^, ^58^, ^59^ ^]^

Optimization strategies aim to reduce these complications and improve stent retention. Techniques include anchoring the stent to adjacent tissues to prevent migration and tailoring the stent size to minimize pressure‐induced injuries. Multi‐disciplinary collaboration among thoracic surgeons, gastroenterologists, and radiologists is crucial during placement and post‐procedure management.^[^ ^28^, ^33^ ^]^ Evidence from clinical studies reinforces the efficacy of stent placement in achieving symptom relief and fistula closure, particularly in cases of malignant TEF.^[^ ^28^ ^]^

#### 4.1.2 Endoscopic Closure and Chemical Electrocoagulation Techniques

**Figure 13:**
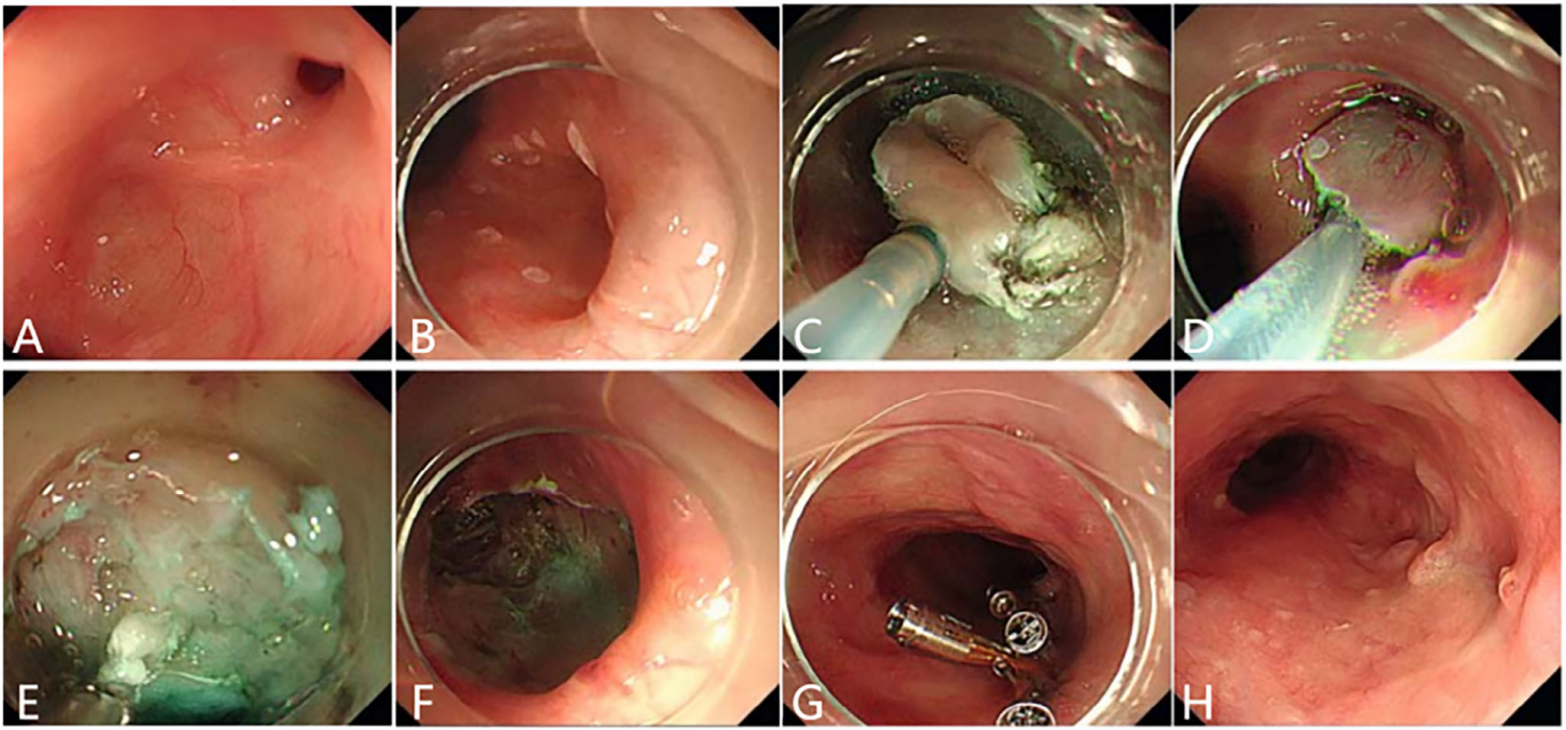
Successful closure of a tracheoesophageal fistula caused by esophageal diverticulum through ESD surgery combined with a titanium clip. A) Endoscopic images showing the endoscopic submucosal dissection procedure. Diverticulum in the right lateral wall of the esophagus and fistulous orifice inside the diverticulum. B) Marking the mucosa surrounding the esophageal diverticulum. C) Dissection of the mucosal and submucosal layers within the diverticulum. D) Excision of mucosal and submucosal layers using a snare trap. E) Dissection of the mucosal patch surrounding the fistulous orifice. F) Part of the muscularis propria inside the diverticulum is cut off. G) Closure of the exposed area using dual‐action tissue clips and SureClips (Micro‐Tech Endoscopy, Inc., Ann Arbor, Michigan, USA). H) Gastroscopy revealed that the tracheoesophageal fistula healed after 3 months. (Reproduced with permission from: Shi L, Long F, Xu H, et al. Chronic tracheoesophageal fistula secondary to esophageal diverticulum successfully treated by endoscopic submucosal dissection and dual‐action tissue clip. Endoscopy. 2023;55(S 01):E1128‐E1130. Georg Thieme Verlag KG. (License: CC BY 4.0)).

Endoscopic closure techniques, such as chemical electrocoagulation, have evolved as minimally invasive alternatives, particularly for recurrent or small congenital and acquired fistulas. These methods employ advanced technologies to close the fistula through localized interventions and avoid open surgery. Closure may involve tissue adhesives or dual‐action clips designed to seal the defect effectively^[^ ^19^, ^48^ ^]^(**Figure** **13**).

Chemical electrocoagulants, such as trichloroacetic acid (TCA), employ a caustic agent that is delivered directly to the fistula site through endoscopic guidance. This approach induces localized inflammation that culminates in tissue fibrosis and fistula closure.^[^ ^19^ ^]^ A study involving a cohort of patients with recurrent TEF demonstrated an impressive fistula closure rate using endoscopic TCA, showing that it is an efficient and safe alternative for patients with contraindications to surgery.^[^ ^19^ ^]^

The advantages of endoscopic techniques include shorter hospitalization, lower morbidity, and rapid recovery, making them particularly appealing for high‐risk patients. Notably, endoscopic closure has seen advancements, such as submucosal dissection techniques and dual‐action tissue clips, which have improved procedural success.^[^ ^48^ ^]^ However, these interventions have limitations, including the potential need for multiple sessions and the challenge of complete closure of larger or more complex fistulas. Julia et al. examined the treatment strategy of 123 patients with TEFs (65 (53%) and 58 (47%) had malignant and benign TEFs, respectively). The initial treatment strategy was nonsurgical (typically esophageal stent placement) in 88 patients (72%), whereas 35 patients (28%) underwent surgical intervention. Both surgical and nonsurgical treatments have demonstrated technical and clinical success in most cases. Notably, patients with malignant TEF who underwent surgical treatment had longer survival than those treated nonsurgically (hazard ratio = 5.6, P = 0.005). In contrast, patients with benign TEF showed similar overall survival rates regardless of whether they received surgical or nonsurgical treatment; reintervention was more common in those who underwent nonsurgical treatment (hazard ratio = 2.3, P = 0.03). Thus, it can be concluded that patients undergoing endoscopic treatment for TEF may face the risk of requiring endoscopic treatment again.^[^ ^60^ ^]^

#### 4.1.3 Endoscopic placement of a new gastrointestinal occluder device for the treatment of malignant TEF

Teng et al. employed a cardiac septal occluder to treat eight patients with TEF. Placement of the cardiac septal occluder was easily and efficiently achieved in all cases. This study demonstrated that cardiac septal occluder therapy in patients with modified TEF could alleviate symptoms, enhance QoL, and improve survival rates, with no significant complications. Thus, the use of a cardiac septal occluder in the management of modified TEF is a safe and effective palliative option.^[^ ^61^ ^]^ Jiang et al. successfully used an atrial septal occluder to close a fistula during esophagogastric anastomosis in a 53‐year‐old patient.; however, 318 days after occluder placement, the patient suddenly developed a severe cough following dilatation of the esophagogastric anastomosis and spontaneously expelled the occluder. The fistula was repaired, and complete closure was confirmed using esophagography. During the 13‐month follow‐up, no recurrence of the fistula was noted.^[^ ^62^ ^]^ This approach may be particularly beneficial for patients at high risk of complications and mortality associated with traditional surgical interventions.

Sang et al. designed a new gastrointestinal occluder device composed of a laminated nitinol mesh featuring two self‐expanding discs connected by a slender waist. This occluder device is lighter on both sides of the umbrella disc, which alleviates pressure on the fistula tissue, thereby reducing the risk of necrosis, bleeding, displacement, shedding, and even suffocation.^[^ ^63^ ^]^ He et al. successfully occluded a 64‐year‐old man's TEF using this new gastrointestinal occluder.^[^ ^64^ ^]^

### 4.2 Surgical Treatment

Although endoscopic treatments are becoming increasingly common, surgical approaches remain indispensable for complex or refractory TEF cases. Surgical repair is particularly relevant for large defects, severe inflammation, and mechanical problems associated with endoscopic failure.

#### 4.2.1 Complex Fistula Repair and Tissue Flap Transplantation

Complex fistula repair often involves meticulous preoperative planning with consideration of the fistula anatomy, patient condition, and surrounding tissue integrity. Traditional surgeries, including direct closure, have high technical demands but offer long‐term relief and successful closure. Preoperative imaging with bronchoscopy or CT helps delineate anatomical features to guide precise surgical planning.^[^ ^18^, ^24^ ^]^

Tissue flap transplantation, which is used to bolster repair sites, is increasingly employed in complex cases. Various tissue flaps, such as muscle, pedicled thymus, or pericardium flaps, reinforce the defect to prevent recurrence and encourage healing.^[^ ^65^, ^66^ ^]^ For example, patients undergoing thoracic or cervical flap‐based repair often exhibit optimal outcomes with minimal postoperative leakage and recurrence rates.^[^ ^24^, ^67^ ^]^

Outcomes are heavily influenced by patient factors, such as age, comorbidities, and extent of tissue damage. Surgical interventions require multidisciplinary follow‐up care, including nutritional and respiratory support to improve patient survival and QoL. Studies have indicated that timely surgical repair yields a high rate of symptom resolution and long‐term tracheoesophageal integrity.^[^ ^67^ ^]^

#### 4.2.2 Innovative Surgery: Double‐layer Repair and Muscle Layer Flap Techniques

Innovative techniques, such as double‐layer repair and muscle‐layer flap transplantation, represent significant advancements in surgical management. Double‐layer repair involves reconstructing the esophageal and tracheal walls independently, thereby reducing the pressure at the repair site and minimizing recurrence.^[^ ^47^, ^66^ ^]^

Muscle flap techniques provide robust, vascularized coverage of the repair site and prevent fistula recurrence, particularly in cases of severe inflammation or scarring. The use of the pectoralis major muscle or other locally available muscle groups has shown improved healing rates and reduced complications.^[^ ^66^, ^67^ ^]^

Comparative studies between conventional and innovative techniques revealed that overlapping repairs, such as double‐layer sutures, exhibited superior outcomes in terms of durability. Innovations in surgical design continue to refine these methods, reduce operative time, and enhance postsurgical recovery.

Surgery may be considered for patients with a large acquired malignant TEF who are in a stable physical condition and have a relatively favorable long‐term prognosis based on tumor histology and stage. However, immediate pre‐operative multidisciplinary discussion is essential.^[^ ^68^ ^]^

### 4.3 Conservative Treatment

Conservative management approaches, which are limited in scope, are essential for managing mild fistulae and stabilizing patients for definitive treatment.

#### 4.3.1 Impact of Nutritional Support on Mild Fistulas

Nutritional support is a cornerstone of conservative TEF management, which aims to optimize a patient's overall health and reduce the risk of complications such as malnutrition and aspiration. Methods include the introduction of enteral nutrition via gastrostomy or jejunostomy, bypassing the fistula and ensuring adequate caloric intake. Evidence suggests that patients with a mild TEF exhibit improved recovery rates when provided with early nutritional intervention. This is particularly crucial for preventing further physiological compromise while facilitating healing.^[^ ^69^ ^]^

#### 4.3.2 Indications and Limitations of Anti‐infection Therapy

Antiinfection therapies are primarily used to manage infections arising from recurrent aspiration or fistula‐related complications. The selection of antimicrobial agents depends on the culturable pathogens and the patient's immune status.^[^ ^28^, ^35^, ^70^ ^]^ However, prolonged antibiotic use has several limitations, including the risk of resistance and adverse systemic effects.

### 4.4 Emerging Treatment Modalities

Advances in regenerative medicine have contributed to the development of novel approaches for TEF management, particularly in refractory or recurrent cases.

#### 4.4.1 Preliminary Applications of Stem Cells and Tissue Engineering in Fistula Repair

Stem cell therapy and tissue engineering represent the frontiers of TEF treatment, focusing on tissue regeneration to close fistulas. Techniques involving scaffold‐based constructs combined with growth factors are promising in experimental models.

Mesenchymal stem cell (MSC) seeding is a promising therapeutic approach for tissue engineering owing to its differentiation potential and proangiogenic, immunomodulatory, and anti‐inflammatory properties. This has been demonstrated in cartilage, bone, and myocardial regeneration.^[^ ^71^, ^72^ ^]^ Findings from a large‐animal study by Petrella et al.^[^ ^73^ ^]^ suggested that the regenerative potential of MSCs in bronchopleural fistulas (BPF) may arise from fibroblast proliferation and collagen matrix deposition.

Kim et al.^[^ ^74^ ^]^ designed an artificial esophagus for rats that could enhance the regeneration of the esophageal mucosa and muscle through the optimal combination of a two‐layered tubular scaffold and an MSC‐based bioreactor system. The regenerated tissues showed that the integration of the esophageal scaffold and its native esophageal tissue was intact and covered with layers of stratified squamous epithelium with several newly developed blood vessels. They created a novel double‐layered scaffold for subsequent studies,^[^ ^75^ ^]^ which served as a tissue‐engineered esophagus created using an electrospinning technique. Before transplantation, human‐derived stem cells were seeded into the lumen of the scaffold, followed by bioreactor cultivation to enhance cellular reactivity. After 3 days of cultivation in the bioreactor system, the tissue‐engineered artificial esophagus was transplanted into a partial esophageal defect (5×3 cm resection) in a canine model. Postoperatively, none of the canine models exhibited signs of leakage or stricture and displayed a normal lumen with complete epithelialization. Significant regeneration of the mucosal layer was confirmed by keratin‐5 immunostaining. Furthermore, alpha‐smooth muscle actin immunostaining revealed markedly greater esophageal muscle regeneration at 12 months than at 6 months.

Jing et al.^[^ ^37^ ^]^ developed a TEF model in beagles to investigate the potential of MSCs in enhancing fistula repair. The results revealed a significant acceleration in the healing process in the MSC‐treated group, with complete fistula closure achieved by three weeks postoperatively. In stark contrast, the TEF group exhibited a fistula that remained considerably patent.

Aho et al.^[^ ^76^ ^]^ described the first‐in‐human application of an autologous MSC‐seeded matrix graft for the repair of multiple recurrent postpneumonectomy BPF. Adipose‐derived MSCs were isolated from the patient's abdominal adipose tissue, expanded, and seeded onto a bioabsorbable mesh surgically implanted at the BPF site. The fistula healed successfully, and the patient remained clinically asymptomatic, with no evidence of recurrence observed during bronchoscopy at 3 months, computed tomography at 16 months, or clinical follow‐up at 1.5 years. They further demonstrated that these patient‐derived MSC populations possessed the potential to differentiate in vitro into several non‐epithelial lineages, which may be necessary for airway regeneration in alignment with the MSC definitions.

Petrella et al.^[^ ^77^ ^]^ transplanted autologous bone marrow‐derived stem cells bronchoscopically to treat a 42‐year‐old male firefighter who developed a bronchopleural fistula following a right extrapleural pneumonectomy for early‐stage malignant mesothelioma. After 60 days of treatment, bronchoscopy revealed complete healing of the resection line, and the orifice that was visible before stem cell implantation had vanished.

Despite certain limitations persisting in our understanding of the specific mechanisms of MSCs in TEF, the study clearly demonstrates the potential for human therapeutic applications. Furthermore, MSCs exhibit heterogeneous immunosafety due to their immune privileges and, more importantly, their immunomodulatory capacities.

#### 4.4.2 Clinical Efficacy of Platelet‐rich Factors

The application of platelet‐rich plasma (PRP) in fistula repair is a novel and minimally invasive intervention. PRP promotes fibroblast proliferation, angiogenesis, and tissue regeneration at repair sites. Preliminary reports have indicated successful fistula closure without surgery in patients with chronic TEF.^[^ ^50^ ^]^

Ongoing research has the potential to integrate these modalities into standard practices and revolutionize TEF management. Wu et al. first used autologous PRP (auto‐PRP) to repair tracheobronchial fistulas (TBF) in humans. Auto‐PRP was injected submucosally around the fistula to promote TBF closure in three patients. The first three injections were administered every 4–7 days to ensure optimal therapeutic effects. Throughout the treatment process, the patients did not require hospitalization; they simply visited the hospital for the scheduled treatment. All three patients involved in this study healed successfully without complications.^[^ ^78^ ^]^

Maxime et al. performed a local injection of autologous PRP around the fistula site in two patients with recurrent, refractory TEF after laryngectomy. After three local injections, the fistula site completely healed after 1–2 weeks of oral feeding.^[^ ^50^ ^]^

Jing et al. injected 10 mL of PRP at multiple points around the fistula site in patients with TEF who could not tolerate metal stent implantation. Three months later, the patient reported that the discomfort in the anterior thoracic region had markedly improved and that he did not choke on liquid food. Gastroscopy revealed that the fistula had healed.^[^ ^46^ ^]^

These case reports highlight the potential benefits of PRP injections as a therapeutic approach for TEFs. However, additional studies involving larger patient populations are required to validate these results. The specific situations for the cases mentioned above are summarized in **Table** **1**.

**Table 1:** Auto‐PRP for repairing TEFs.

cases	age	sex	Cause of Disease	Type of Fistula	Number of Injections	First Injection to Fistula Healing Time
case1	51	male	After right lower lobectomy for adenocarcinoma of the lung	TPF	2	17weeks
case2	69	male	left lower lobectomy for left lung multiple abscesses	TPF	6	15weeks
case3	55	male	right upper lobectomy for right upper lung fungal infection	TPF	4	17weeks
case4	64	male	functional pharyngo‐laryngectomy	TEF	2	3weeks
case5	58	male	tracheoesophageal puncture	TEF	3	17weeks
case6	70	male	Thoracoscopic and laparoscopic resection of esophageal cancer	TEF	2	3weeks

TBF: tracheobronchial fistula;TEF: tracheoesophageal fistula

## 5 Analysis of Treatment Outcomes and Complications

### 5.1 Efficacy Evaluation

#### 5.1.1 Comprehensive Analysis of Cure Rates and Recurrence Rates

“Cure” in the treatment of TEF entails the successful closure of the abnormal connection between the trachea and esophagus, leading to the resolution of related symptoms and prevention of recurrence. Currently, endoscopic and surgical interventions are the primary treatment modalities. A recent retrospective analysis comparing the outcomes of endoscopic and surgical repairs over 10 years demonstrated that surgical repair achieved definitive closure in most cases (75%), with relatively low recurrence rates in experienced surgical centers. Endoscopic treatments have shown success rates of ≈50%, often necessitating additional interventions due to complications such as incomplete closure or stent migration.^[^ ^24^ ^]^

Factors influencing cure rates include the etiology of the fistula (congenital or acquired) and patient demographics, such as age, comorbidities, and complexity of the fistula. For instance, congenital TEFs corrected using thoracoscopic surgery have demonstrated high success rates, particularly in experienced pediatric surgical centers. Studies on Gross Type C EA with a distal fistula corroborate these findings, with surgical repair yielding satisfactory outcomes; however, recurrence, particularly in complex cases, necessitates individualized follow‐up care.^[^ ^21^, ^49^ ^]^ For H‐type TEFs, endoscopic techniques have shown moderate efficacy, often requiring adjunct tissue grafts or improvements in repair strategies to enhance outcomes.^[^ ^11^, ^49^ ^]^

Recurrence after repair remains a significant concern, particularly in surgically treated cases of complex malformations or with concurrent inflammatory conditions. Recurrence rates in repaired TEFs range from 10% to 15%, depending on the location and size of the fistula, surgical expertise, and the presence of underlying factors, such as severe gastroesophageal reflux (GER). GER appears to significantly increase the likelihood of recurrence; thus, antireflux measures are crucial adjunctive treatments.^[^ ^49^, ^79^ ^]^ Innovative techniques such as interpositional grafts using muscle flaps or synthetic materials have proven effective in reducing recurrence rates.^[^ ^24^, ^80^ ^]^

#### 5.1.2 Studies on Survival and QoL Improvements

Survival rates for TEF patients with TEFs have improved significantly over the past decade owing to advancements in diagnostic and repair techniques. Longitudinal studies have reported survival rates exceeding 90% in high‐resource settings, with major determinants being early diagnosis and timely surgical intervention.^[^ ^22^, ^49^ ^]^ However, acquired TEFs, particularly those resulting from malignancies or radiation therapy, are associated with poor survival outcomes. For instance, treatment of malignant TEF with stents shows a higher median survival than conservative therapy, although it is still limited by disease progression.^[^ ^28^ ^]^

Post‐treatment QoL improvements are multidimensional and involve factors such as the resolution of dysphagia, prevention of pulmonary infections, and nutritional status optimization. Various tools, including EQ‐5D and SF‐36, have been used to quantify these improvements. Patients undergoing endoscopic or minimally invasive surgical procedures report quicker recovery times and better short‐term QoL scores than those undergoing open surgeries.^[^ ^24^, ^69^ ^]^ Enhanced recovery protocols incorporating nutritional rehabilitation and GER management have further contributed to sustained improvements in QoL metrics.^[^ ^66^, ^69^ ^]^

Studies have also highlighted that innovative treatments, such as local PRP^[^ ^50^ ^]^ or MSCs^[^ ^78^ ^]^, used to promote fistula healing, have led to promising outcomes in selected patients. Such noninvasive methods have shown effective closure with minimal complications, reducing the need for major interventions that might negatively impact QoL.^[^ ^46^, ^50^ ^]^ In malignant TEFs, combined approaches using stents and adjunct therapies have positively influenced patient‐reported outcomes, emphasizing symptom control and prolongation of life expectancy.^[^ ^28^ ^]^

### 5.2 Complications Analysis

#### 5.2.1 Endoscopic‐related Complications: Stent Displacement and Local Infection

Complications related to endoscopic treatment predominantly involve stent‐related issues and localized infections. Stent displacement, particularly in cases where self‐expanding metallic stents (SEMS) are used, occurs in ≈10%–20% of patients.^[^ ^28^, ^33^ ^]^ Risk factors for displacement include improper stent positioning, excessive mobility at the fistula site, and insufficient anchoring. Technological advancements in stent design, such as modifications to provide greater stability, have reduced this risk to some extent.^[^ ^33^ ^]^

Local infections manifesting as abscesses, granulation tissue formation, or fistula site sepsis are another significant concern. Studies have reported infection rates of 15%–35%, with higher incidences in patients with pre‐existing pulmonary infections or immunosuppressed states.^[^ ^28^, ^65^ ^]^ Management typically involves antibiotic therapy, debridement, or stent revision, depending on the severity of the infection.

New strategies to address these complications include the use of endoscopic adjunct therapies, such as PRP injections, which not only aid healing but also diminish infection risks by promoting localized vascular regeneration.^[^ ^46^, ^50^ ^]^ Additionally, some centers advocate early stent removal and replacement protocols to preemptively address infections and minimize displacement risks.^[^ ^28^ ^]^

#### 5.2.2 Surgical Complications: Anastomotic Recurrence, Strictures, and Injury

Surgical repair of TEFs is associated with complex challenges, including anastomotic recurrence, stricture formation, and iatrogenic injury to adjacent structures. Anastomotic recurrence remains a significant complication, particularly in patients with significant inflammatory responses or poor tissue quality. The incidence rates range from 10% to 15%, with many cases necessitating re‐intervention via minimally invasive or open surgical techniques.^[^ ^22^, ^80^, ^81^ ^]^ The mechanisms underlying recurrence often involve inadequate suture approximation, poor blood supply at the anastomotic site, or post‐repair mechanical stress. Prophylactic measures, including the use of interpositional flaps or reinforced suturing, have shown positive outcomes in reducing recurrence.^[^ ^24^, ^81^ ^]^

Strictures at the repair site are a frequent complication, occurring in ≈20%–30% of patients following the surgical repair of congenital or acquired TEFs.^[^ ^21^, ^22^ ^]^ The contributing factors include excessive scarring, GER, and tension at the anastomotic site. Early detection through routine endoscopic surveillance allows for timely management using balloon dilation or topical anti‐inflammatory therapies to prevent stricture progression.^[^ ^81^ ^]^

Injuries to critical structures, such as the recurrent laryngeal nerve and adjacent vasculature, are of particular concern in high‐risk surgical cases. Studies have reported postoperative recurrent laryngeal nerve paresis rates of over 30% in some pediatric repairs, with varying recovery outcomes depending on intraoperative neuroprotection measures.^[^ ^24^, ^49^ ^]^ Real‐time neuromonitoring during surgery reportedly reduces nerve injury rates and facilitates safe surgical planning.^[^ ^24^ ^]^

In summary, although both endoscopic and surgical approaches present noteworthy complications, advances in procedural techniques, adjunct therapies, and postoperative management strategies continue to improve patient outcomes. Sustained innovations in the stent design, biomaterials, and minimally invasive procedures are expected to mitigate the risks associated with these treatments.

## 6 Multidisciplinary Collaboration Framework and Practical Applications

### 6.1 Importance of Multidisciplinary Team Collaboration

In the management of TEFs, the integration of various specialties into a cohesive multidisciplinary approach is critical for optimizing patient outcomes. Patients with TEFs often present with complex clinical conditions that require precise and timely intervention across multiple domains. Effective collaboration enables better diagnostic accuracy, tailored treatment strategies, and the prevention of complications, thereby addressing the multifaceted challenges associated with this condition.

#### 6.1.1 Collaborative Practices among Thoracic Surgery, Gastroenterology, and Radiology

Thoracic surgeons form a cornerstone in the treatment of TEFs, focusing on surgical repair of the fistula and its associated anatomical abnormalities. Their approach often requires preoperative input from gastroenterologists who handle the gastrointestinal aspects of the disease, including nutritional assessment, management of reflux, and assistance in interpreting the endoscopic findings. Gastroenterologists may also play an essential role in postoperative follow‐up, particularly in addressing complications such as esophageal strictures or residual fistulas.

Radiologists contribute equally by providing high‐resolution imaging and diagnostic precision for both initial assessments and subsequent evaluations. Techniques such as CT and esophagography allow detailed visualization of the fistula anatomy, thereby assisting surgeons in strategic planning. The integration of expertise ensures accurate diagnosis and minimizes the misinterpretation of imaging results, which could otherwise delay appropriate interventions.^[^ ^82^, ^83^, ^84^ ^]^

Regular interdisciplinary meetings and case conferences are indispensable for complex cases such as congenital TEF associated with vertebral defects, anorectal malformations, and cardiac anomalies. One notable example is the coordination involved in managing patients with the VACTERL association, in which conditions coexist, and tailored plans must be developed. A unified team comprising thoracic surgeons, gastroenterologists, and radiologists facilitates comprehensive treatment while avoiding overlapping responsibilities and miscommunication.^[^ ^84^ ^]^

#### 6.1.2 Core Roles of Nutrition and Physical Rehabilitation in Patient Management

Nutrition plays a vital role in the recovery of patients with TEFs. Malnutrition is a pervasive concern, particularly in cases in which the TEF impairs the ability to swallow or leads to recurrent aspiration. Dietitians integrated into the multidisciplinary team assessed the caloric needs and oversaw enteral or parenteral nutrition regimens, ensuring that patients received adequate nourishment preoperatively and postoperatively. This is particularly critical in cases of advanced disease, where systemic cachexia may exacerbate the clinical scenario.^[^ ^82^, ^83^ ^]^

Physical rehabilitation is another crucial component, particularly in adults, where respiratory complications pose significant challenges. Respiratory therapists design individualized programs to improve lung function and minimize complications, such as atelectasis. Physical therapists complement recovery by implementing structured exercises that enhance mobility, strength, and endurance, particularly for patients with chronic diseases.

Incorporating nutrition and physical rehabilitation specialists into a multidisciplinary team adds significant value to patient‐centered care. These professionals contribute to the overall treatment by addressing aspects beyond primary surgical repair, thereby enhancing the recovery and long‐term QoL of the patients.^[^ ^84^ ^]^

### 6.2 Implementation Challenges and Solutions

Despite its numerous benefits, the implementation of a multidisciplinary collaborative framework for TEF management faces significant hurdles. Challenges such as fragmented communication, difficulties in data integration, and logistical issues in surgical planning often hinder seamless execution. Addressing these challenges requires strategic solutions to improve coordination and efficiency.

#### 6.2.1 Information Integration in Multidisciplinary Diagnostic Processes

One of the foremost barriers in a multidisciplinary setting is the lack of efficient data sharing among team members from different specialties. Misaligned reporting formats or delays in transferring medical information can lead to suboptimal decision‐making and treatment delays. For example, incomplete diagnostic imaging reports or inconsistent endoscopic findings may result in delayed surgical intervention and worsened patient prognosis.^[^ ^83^ ^]^

One promising solution is the adoption of shared electronic health records and collaborative software platforms.^[^ ^85^, ^86^ ^]^ When implemented appropriately, electronic health records have been shown to improve the quality and reliability of healthcare service delivery. These technologies allow real‐time access to patient information across departments, thereby fostering integrated diagnostic and treatment planning. In TEF cases, the use of standardized protocols for imaging, endoscopy, and surgical findings ensures transparency and minimizes the risk of miscommunication. Customizable care pathways designed specifically for TEF can further streamline information exchange and facilitate adherence to clinical guidelines.^[^ ^83^ ^]^

In addition, regular interdisciplinary meetings and workshops focused on protocol standardization help align objectives, ensuring that diagnostic and therapeutic recommendations are cohesive and actionable. This systematic approach minimizes uncertainty and allows for dynamic adjustments in treatment strategies as patient conditions evolve.^[^ ^84^ ^]^

#### 6.2.2 Surgical Planning and Postoperative Patient Management

Surgical planning for patients with TEFs, particularly those with secondary complications from malignancies or the VACTERL association, requires input from several specialties to ensure that all aspects of the condition are addressed. The misalignment of these inputs can lead to incomplete surgical repair or unforeseen post‐operative complications. For example, complex fistula repairs may require precise preoperative imaging, nutritional stabilization, and a post‐operative ventilation strategy, all of which require careful inter‐specialty coordination.^[^ ^26^, ^68^ ^]^

Multidisciplinary preoperative assessment meetings are instrumental for overcoming these challenges. In addition to conventional imaging reviews, a thorough analysis of the nutritional and respiratory needs helps ensure that the patient is optimized for surgery. These meetings provide a platform for specialists to share insights, clarify responsibilities, and anticipate complications, resulting in a unified surgical strategy.^[^ ^87^ ^]^

Postoperative management adds another layer of complexity, particularly when addressing ongoing fistula‐related issues. For instance, TEF resulting from malignancies often requires close surveillance for recurrence or progression, combined with follow‐up by thoracic surgeons, gastroenterologists, and oncologists. Integrating physiotherapists into this stage of patient care enables targeted rehabilitation to address specific deficits such as respiratory weakness or mobility restrictions.^[^ ^26^ ^]^

Furthermore, the establishment of follow‐up protocols shared across disciplines can minimize gaps in patient management. Standardized schedules for imaging, nutritional evaluation, and physical assessment provide continuous oversight, ensuring that identification and management are managed promptly.^[^ ^84^, ^88^ ^]^

## 7 Future Research Directions

### 7.1 Potential of New Medical Technologies

#### 7.1.1 3D Printing Technology in Developing High‐fidelity Surgical Simulators

3D printing allows for the creation of accurate patient‐specific anatomical models derived from imaging techniques such as MRI or CT scans. These models can help surgeons visualize individual variations in the anatomy and plan for the intricacies of surgical procedures more effectively. 3D printing technology presents new opportunities for personalized and precise interventions to repair TEFs. By facilitating the creation of customized implants and scaffolds that align closely with a patient's unique anatomy, 3D printing enhances surgical precision. This technology enables the development of patient‐specific models that aid in preoperative planning, allowing surgeons to visualize complex anatomical relationships and tailor their strategies, thereby increasing the likelihood of successful outcomes. Moreover, innovative biodegradable materials combined with 3D printing offer the potential for implants that promote tissue regeneration and natural degradation over time. This approach may reduce the long‐term risks associated with permanent foreign materials, such as infection or rejection.^[^ ^16^, ^89^ ^]^

With the advancement of minimally invasive surgical techniques, thoracoscopy has emerged as the preferred approach for repairing EA/TEFs. Compared with traditional open chest surgery, thoracoscopy offers advantages such as reduced trauma and quicker recovery.^[^ ^90^ ^]^ Nevertheless, thoracoscopic repair of EA/TEF presents significant technical challenges, particularly in neonatal patients in whom the surgical space is confined, tissues are delicate, and precision is paramount.^[^ ^91^ ^]^ Studies have indicated that even seasoned surgeons may encounter complications during thoracoscopic EA/TEF repair, including extended operation times and anastomotic leaks during thoracoscopic EA/TEF repair.^[^ ^90^ ^]^ Thus, enhancing the technical proficiency of surgeons is an urgent issue that requires resolution.

In recent years, the use of 3D printing technology in the development of high‐fidelity surgical simulators has provided innovative solutions to this challenge. By leveraging 3D printing, surgical models that closely mimic actual anatomical structures can be created, thereby offering surgeons a safe and consistent training environment.^[^ ^91^, ^92^ ^]^ For instance, Zahradnikov et al. developed an EA/TEF surgical simulator utilizing 3D printing and silicone casting, and validated its effectiveness through Objective Structured Technology Evaluation (OSATS).^[^ ^91^ ^]^ The research findings revealed that the simulator significantly distinguishes between task fluency and overall skill evaluation in minimally invasive surgery, particularly highlighting the differences between novices and experts.^[^ ^85^ ^]^ Additionally, the 3D printed thoracoscopy EA/TEF surgical simulator developed by Youn et al. has garnered high praise from young surgeons, achieving a global rating of 3.38/4, underscoring its practicality and effectiveness in training.^[^ ^92^ ^]^

To objectively assess the technical proficiency of surgeons, researchers have started integrating motion tracking and force measurement techniques into the design of surgical simulators. These technologies enable the recording of movement paths and operating forces of surgical instruments, thereby providing quantitative data for technical evaluations.^[^ ^93^, ^94^ ^]^ For instance, Choi et al. demonstrated through motion tracking that experts exhibited significantly shorter instrument movement paths in EA/TEF repair tasks than novices, indicating greater operational efficiency.^[^ ^93^ ^]^ Additionally, Liddy et al. discovered that the tension applied by experts during esophageal anastomosis tasks is more consistent and that excessive force occurrences are notably reduced by incorporating force sensors in a 3D‐printed simulator.^[^ ^94^ ^]^ These findings suggest that motion tracking and force measurement techniques not only differentiate surgeons based on their experience levels but also facilitate targeted feedback for technical training.

The development of high‐fidelity surgical simulators is a significant advancement in the field of surgical training. By integrating 3D printing technology with patient‐specific imaging data, researchers can create highly realistic surgical models that replicate intricate anatomical structures and surgical environments.^[^ ^95^, ^96^ ^]^ For example, Moorhead et al. developed an EA/TEF repair simulator featuring motion‐ and force‐tracking components that achieved remarkable sensor accuracy and precision.^[^ ^95^ ^]^ Hong et al. developed a reusable thoracoscopic surgery simulator utilizing CT data from pediatric patients, which exhibited mechanical properties and anatomical accuracy comparable to those of actual tissues.^[^ ^96^ ^]^ These simulators offer a safe and effective training platform for surgeons and provide a scientific foundation for technical evaluation and the design of training courses.

Despite the significant advancements in 3D printing and simulation technology for EA/TEF surgical training, several challenges and directions for future development remain. First, the cost and technical complexity of simulators may hinder their widespread adoption, necessitating further optimization of the design and manufacturing processes to lower costs and enhance accessibility.^[^ ^91^, ^92^ ^]^ Second, the evaluation of the effectiveness of simulators requires large‐scale multicenter studies to validate their practicality and training effectiveness in real surgical settings.^[^ ^93^, ^94^ ^]^ Future research should focus on integrating simulator training with clinical practice to maximize training outcomes and improve patient prognosis.^[^ ^95^, ^96^, ^97^ ^]^

In summary, the long‐term management of EA/TEF and the technical challenges associated with thoracoscopic surgery have introduced new demands for surgical training. By integrating 3D printing technology, motion tracking, and force measurements, high‐fidelity surgical simulators offer surgeons safe and effective training platforms. These advancements are anticipated to further enhance the standardization and optimization of EA/TEF surgical techniques.

#### 7.1.2 Application Prospects of 3D Printing and Biodegradable Materials for EA/TEF

In tracheal tissue engineering, constructing biomimetic tracheal scaffolds with adequate mechanical properties and biological functions remains a significant challenge. The natural trachea features a C‐type cartilage ring and a complex inner wall that serves diverse functions. To replicate this structure, researchers have employed mold casting and electrospinning techniques to fabricate biomimetic tracheal stents made from silk fibroin and polycaprolactone. The C‐type cartilage ring of the scaffold was affixed to the outer wall of a silk fibroin tube via electrospinning, resulting in a fibrous scaffold with a porous architecture. In vitro enzymatic hydrolysis experiments revealed that silk fibroin underwent substantial degradation within one week, whereas the degradation rate of polycaprolactone was comparatively slower. Moreover, compression tests demonstrated that the compressive strength of the stent exceeded that of the natural rabbit trachea. Subsequent subcutaneous transplantation studies have indicated that as the material degrades, fibrous tissue and blood vessels gradually proliferate, enhancing the blood supply and providing improved mechanical properties to the stent.^[^ ^98^ ^]^ However, a limitation of this study was that the fully pre‐vascularized biomimetic scaffold had not been subjected to tracheal transplantation to promote the differentiation of ciliated cells in vivo. Additionally, the scaffold's thickness, exceeding that of the rabbit trachea, hindered its robustness for secure attachment to the tracheal suture. This challenge will be the focus of the team's next research endeavor.

Several pilot studies have demonstrated the application of these principles in related fields. For instance, customized 3D‐printed scaffolds have been used to repair defects in esophageal and tracheal tissues with initial success in preclinical models. These frameworks, combined with bioactive coatings such as growth factors or stem cell seeding, accelerate healing by fostering cellular proliferation and tissue regeneration.^[^ ^37^ ^]^ In addition, biodegradable implants have been engineered for temporary structural support, allowing natural tissue remodeling before gradually dissolving, thereby reducing the need for additional surgical interventions.^[^ ^89^ ^]^

However, there are several challenges in the implementation of 3D printing and biodegradable materials for TEF treatment. Regulatory and ethical considerations are significant barriers because the production and clinical use of these materials must adhere to rigorous standards to ensure patient safety. In addition, the compatibility between biomaterials and human tissues, durability under physiological conditions, and potential inflammatory or immune responses are areas of ongoing investigation. Moreover, because the TEF is a rare and complex condition, the development of standardized solutions requires extensive multidisciplinary collaboration. Future research should focus on optimizing the biocompatibility of materials, enhancing their mechanical properties, and evaluating long‐term patient outcomes in controlled clinical trials.^[^ ^37^, ^50^ ^]^

#### 7.1.3 Innovative Intraoperative Localization Methods for Esophageal Atresia/Tracheoesophageal Fistula

The application of innovative intraoperative localization methods has significantly enhanced surgical precision and efficiency. For example, Hua K et al.^[^ ^99^ ^]^ have used a combination of thread guidewire and indocyanine green (ICG) fluorescence to assist in the localization of rTEF, resulting in highly favorable outcomes. The specific application method as follows:

Under general anesthesia, the surgical team inserted a guidewire from the tracheal aspect of the fistula into the esophagus guided by bronchoscopy. The guidewire was then extracted from the esophagus or stomach, forming a ‘U’ loop, with a silk thread tied to its end. Subsequently, the guidewire was replaced by the silk thread, and the ends of the thread were secured at the corner of the mouth. This method enables precise intraoperative localization of the fistula, allowing the surgeon to pull the thread for guidance.

During bronchoscopy, the surgeon administered 0.5 ml of ICG (at a concentration of 0.125 mg/ml) directly into the fistula when the orifice was visible. If the orifice could not be found, ICG was sprayed via esophagoscopy. The patient then underwent thoracoscopic repair utilizing a fluorescence imaging thoracoscopy system (DPMENDOCAM‐01). Initially, the surgeon separated pleural adhesions under natural light. After exposing the esophagus, the fluorescence imaging mode was activated, clearly revealing the location of the fistula.

A total of 106 patients were included in this study, with all cases categorized based on whether using localization techniques, resulting in the localization group (*n* = 52) and the nonlocalization group (n = 61). The median operation time in the localization group (2.5 h) was significantly lower than in the non‐localization group (3.0 h) (P = 0.001). Additionally, the average postoperative hospital stay was significantly shorter in the localization group (17.7±7.5 days) than in the nonlocalization group (23.6±20.0 days) regarding the fistula type of TEF (P = 0.03).

This study faces certain limitations, primarily due to its single‐center design, which raises questions about the generalizability of the findings. While the results are significant within the institution, they may not be easily replicated in other centers or under varying conditions. Unique patient demographics, healthcare practices, and local protocols could influence outcomes, thereby restricting the broader applicability of the conclusions. To fully validate these findings, further confirmation through multicenter randomized trials is essential, as these would provide more robust evidence across diverse patient populations and healthcare environments.

### 7.2 Upgrade of Personalized Treatment Models

#### 7.2.1 Genomic‐based Treatment Optimization Strategies

Personalized medicine, particularly when informed by genomic data, has emerged as a vital component in optimizing treatments for complex medical conditions, including TEFs. Genomic analysis enables the identification of patient‐specific factors that may influence healing or therapeutic response, providing a foundation for tailored interventions. For instance, Jing et al. discovered that treatment with MSC supernatant significantly reduces the expression of pro‐inflammatory markers, including p–NF–κB, TLR4, IL‐1β, and TNF‐α, thereby mitigating the pathological inflammatory response in TEF. This effect primarily results from a decrease in the abundance of M1 macrophages and their pro‐inflammatory activity. Variations in genes linked to inflammatory responses or tissue remodeling could guide the development of customized surgical or pharmacological approaches to enhance recovery.^[^ ^37^ ^]^These findings may eventually translate into more precise treatment plans, such as selecting specific biocompatible materials for repair or tailoring anti‐inflammatory regimens to minimize postoperative complications.

Wild K et al.^[^ ^100^ ^]^ conducted a retrospective cohort study involving 212 infants with EA/TEF revealed a complex interplay of genetic and environmental factors contributing to TEF. Genetic testing, including karyotype analysis, chromosomal microarray analysis, single‐gene testing for CHD7, and exome sequencing, demonstrated diagnostic yields ranging from 3% to 63%, depending on the testing modality and patient phenotype.

Notably, genome‐wide testing, such as exome or genome sequencing, was recommended over chromosomal microarray testing due to its superior diagnostic yield. For instance, Sy M et al.^[^ ^101^ ^]^ found that in complex or syndromic cases, exome sequencing identified genetic abnormalities in 8 out of 37 patients, underscoring the potential of advanced genomic testing to enhance prognostication and facilitate individualized management. Despite these potential benefits, exome sequencing is not universally recommended for individuals with EA/TEF lacking a molecular diagnosis. This may stem from uncertainties regarding the effectiveness of exome sequencing in this population.

Khattar D et al.^[^ ^102^ ^]^ recommend that for patients with EA/TEF, rapid exome/genome sequencing (ES/GS) represents the most effective genetic test in the neonatal intensive care unit (NICU) for obtaining a timely diagnosis. However, this testing is not widely accessible and may incur significant costs at certain institutions and healthcare systems. In such cases, particularly when a distinct phenotype is not identifiable, they suggest initiating the diagnostic process with a microarray and a gene panel that includes genes known to be associated with EA/TEF (such as CHD7, SOX2, EFTUD2, MYCN, FANCA, FANCB, FANCC, FGFR3, NRXN1, TCF4), with the option to subsequently perform ES/GS if initial results are inconclusive. By thoroughly characterizing the phenotype and genotype of the EA/TEF patient in the NICU, a comprehensive care plan can be formulated, providing parents with enhanced insight into their child's healthcare needs and anticipated outcomes regarding nutritional and medical milestones. Further research is essential to fully elucidate the genetic etiology of this complex birth defect, thereby improving our understanding of genotype‐phenotype relationships and enabling better predictions of long‐term outcomes.

While the exact genetic contributors to TEF are not fully understood, Sy M et al. have highlighted the role of specific genetic mutations and chromosomal abnormalities in its development. For instance, mutations in genes such as CHD7, SOX2, and FOXF1 have been implicated in the pathogenesis of TEF, often in the context of syndromes like CHARGE syndrome and VACTERL association. These genetic insights provide a deeper understanding of the molecular mechanisms underlying TEF and may guide personalized management strategies. The integration of genomic insights with advanced therapeutic strategies is crucial for optimizing the management of TEF. Understanding the genetic contributors to TEF can aid in the identification of patients at risk for associated anomalies, guiding personalized treatment decisions. The next step could involve identifying preventive strategies for EA development, akin to the prevention of some neural crest deformities by folic acid supplementation.^[^ ^8^, ^9^ ^]^ Furthermore, the development of novel therapeutic technologies and devices is essential for addressing the complex and often inoperable nature of TEF, ultimately enhancing patient outcomes and long‐term prognosis.^[^ ^101^ ^]^

#### 7.2.2 Analysis of Treatment Methods for TEF in Children and Adults

TEF in children primarily manifests as congenital EA and TEF and is typically diagnosed in newborns. Surgical interventions, including thoracoscopy and open chest surgery, remain the cornerstone of treatment.

Zhao et al. conducted a retrospective analysis of the clinicopathological features of Type D EA with TEF. Among 386 EA/TEF cases, 14 (3.6%) were identified as type D EA/TEF. Preoperative diagnosis of proximal TEF was established in only two patients, while seven were diagnosed intraoperatively. Initial surgical misdiagnosis occurred in five cases, which were later confirmed via bronchoscopy. Neonatal one‐stage repair of proximal and distal TEF, performed through thoracoscopy or thoracotomy, was done in seven patients. Due to missed diagnosis and other reasons, the remaining seven patients underwent two‐stage surgery for proximal TEF repair, involving cervical incision and thoracoscopy. Postoperative complications, including anastomotic leakage, pneumothorax, esophageal stricture, and recurrence, were observed in 10 of the 14 patients. Neonates undergoing one‐stage repair of distal and proximal TEF demonstrated a significantly higher anastomotic leak rate (4/7 cases) than their counterparts receiving two‐stage proximal TEF repair, in which only one in seven patients experienced this complication.^[^ ^103^ ^]^

Yang et al. conducted a retrospective analysis of 190 patients to compare the clinical outcomes of the thoracoscopic approach and thoracotomy surgery in patients with gross type C EA and TEF. Research has shown that, to a certain extent, thoracoscopic surgery reduces the incidence of anastomotic leakage and increases the incidence of anastomotic strictures. However, there were no statistically significant differences between the two groups in terms of operative time, postoperative pneumothorax, anastomotic leakage, anastomotic stricture, and recurrent TEF, indicating that thoracoscopic surgery for gross type C EA/TEF is a safe, effective, and minimally invasive technique with a comparable operative time and incidence of postoperative complications.^[^ ^21^ ^]^

Yang et al. conducted a meta‐analysis of eight observational clinical studies involving 452 children. The meta‐analysis of two major postoperative complications (leaks and strictures) did not show significant differences between thoracoscopic repair (TR) versus conventional open repair (COR). Although associated with a longer operative time, TR has the advantages of an earlier time‐to‐extubation and 1st oral feeding and a shorter hospital stay.^[^ ^104^ ^]^

Hua et al. retrospectively analyzed the clinical characteristics and outcomes of 103 consecutive children who underwent thoracoscopic surgery for recurrent TEF (rTEF) performed by a single surgeon after EA/TEF repair at two different institutions. This study aimed to explore the safety and efficacy of thoracoscopic treatment for rTEF. Primary surgeries were performed via thoracoscopy (*n* = 75; 72.82%) or open surgery (*n* = 28; 27.18%). After rTEF repair, the incidences of esophageal leakage, esophageal stricture, and TEF recurrence were 12.8%, 33.4%, and 10.8%, respectively. After follow‐up, 87 patients survived, 6 died, and 10 were lost to follow‐up. The results of thoracoscopic surgery for rTEF were comparable to those of previously reported thoracotomy surgeries. Owing to the clear field during the operation, rapid patient recovery, and esthetic results, the thoracoscopic approach may be a better choice for experienced pediatric surgeons.^[^ ^22^ ^]^

Friedmacher et al. retrospectively analyzed the temporal changes in morbidity and mortality in 109 children with EA/TEF treated at a tertiary‐level center, focusing on postoperative complications and their impact on long‐term gastroesophageal function. Patients may present postoperative complications, such as anastomotic leakage, anastomotic stricture, and esophageal dysmotility. Patients with dysphagia requiring endoscopic foreign body removal, esophagitis, and Barrett's esophagus identified after EA/TEF repair are common and should be expertly managed to reduce the risk of long‐term morbidity. Regular multidisciplinary surveillance with transitional care into adulthood is recommended for all patients with EA/TEF.^[^ ^105^ ^]^

Okuyama et al.^[^ ^90^ ^]^ conducted a study involving 64 patients with type C EA, all of whom underwent thoracoscopic repair surgery performed by young pediatric surgeons during training. The results indicated that all patients underwent successful correction via minimally invasive surgery (MIS) 3 days after birth. The median duration of surgery was 181 min (range, 127–334 min). Nasogastric tube feeding was initiated on the first postoperative day, followed by oral feeding 6 days post‐operation. Postoperative complications included leakage (14.1%), stenosis (51.1%), and recurrent TEFs (7.8%). This suggests that thoracoscopic repair of the EA can be performed safely, yielding favorable outcomes while providing the advantages of MIS. However, the procedure remains challenging and should be conducted exclusively in pediatric centers with extensive MIS experience, particularly when training young pediatric surgeons. These centers must have access to a multidisciplinary team, including neonatologists, pediatric anesthesiologists, surgeons, and ENT specialists, to ensure optimal care for hemodynamic and respiratory support, as well as gastrointestinal and developmental outcomes.

Acquired TEF is more commonly observed in adults, mostly due to malignancy, trauma, or post‐therapeutic complications such as intubation or radiotherapy.^[^ ^2^, ^3^ ^]^

As adult organs become fully developed, concerns about ongoing growth become unnecessary, thereby expanding the range of viable treatment options. For example, the placement of covered metallic stents has become a critical option for TEF management, particularly in malignant or postoperative cases. Self‐expandable stents, commonly made of materials such as nitinol or stainless steel, are often favored because of their flexibility and durability.^[^ ^28^ ^]^ Endoscopic closure methods, including chemical electrocoagulation paired with tissue adhesives or dual‐action clips, can effectively treat recurrent or small congenital or acquired fistulas, thereby eliminating the need for open surgery.^[^ ^19^, ^48^ ^]^ Endoscopic placement of a novel gastrointestinal occluder device is a relatively new treatment option. Although the sample size of this study was limited, the treatment outcomes were satisfactory. This approach may be particularly beneficial for patients at high risk of complications and mortality associated with traditional surgical interventions.^[^ ^61^, ^62^, ^63^ ^]^

Although endoscopic treatments are becoming increasingly common, surgical approaches remain indispensable for complex or refractory TEF cases. Surgical repair is particularly relevant for large defects, severe inflammation, and mechanical problems associated with endoscopic failure. Examples include complex fistula repair, tissue flap transplantation,^[^ ^24^ ^]^ as well as double‐layer repair and muscle layer flap techniques.^[^ ^68^ ^]^ Before performing these surgical procedures, multidisciplinary discussions are essential.

Conservative treatment is essential in the management of adult TEF. This approach includes providing nutritional support to patients with mild TEFs or stabilizing their overall condition to facilitate further treatment^[^ ^69^ ^]^. It also encompasses antiinfective treatments for infections resulting from recurrent issues or complications related to fistulas.^[^ ^28^, ^70^ ^]^ Conservative treatment also involves innovative methods such as the injection of MSCs and PRP around the fistula site, which have demonstrated notable therapeutic effects.^[^ ^75^ ^]^

## 8 Conclusion

### 8.1 Summary of Current Progress in Treatment of TEF

Significant advancements have been achieved in the management of TEF through both surgical and non‐surgical approaches. Surgical techniques, particularly thoracoscopic and open procedures, have seen notable refinements over time. The application of innovative intraoperative localization methods like thread guidewire and ICG fluorescence has significantly enhanced surgical precision and efficiency.

Non‐surgical treatments, including endoscopic techniques, show promise in selected TEF cases. Covered metallic stent placement is gaining traction for its efficacy in sealing fistulas while minimizing invasive interventions. Furthermore, approaches such as endoscopic closure techniques and chemical electrocoagulation have provided alternative avenues for managing complex or recurrent cases. Conservative therapies, including nutritional support and anti‐infection strategies, remain integral to symptom management and enhance patient recovery, particularly in individuals with mild or less complex fistulas.

These collective advancements have contributed to improved cure rates, reduced recurrence rates, and enhanced overall QoL for TEF patients with TEF. Emerging modalities, such as tissue engineering and stem cell‐based therapies, have also opened new frontiers, offering potential solutions for cases with difficult anatomical or pathological conditions. As the field evolves, novel approaches, as evidenced by ongoing research, continue to push the boundaries of what is possible in TEF treatment.

In summary, although substantial progress has been made, the incorporation of cutting‐edge surgical techniques, consolidated endoscopic strategies, and innovative conservative measures provides a robust framework for effectively addressing the complexities of TEF management. Nevertheless, there is scope for further improvements and innovations, particularly in addressing recurrent and treatment‐resistant cases.

Although many signaling pathways are promising therapeutic targets, their clinical translation is challenged by issues such as targeting specificity, potential side effects, and intricate interactions among various pathways. Future research should prioritize combinatorial targeting strategies and personalized approaches tailored to the distinct pathophysiology of fistulas in individual patients.

Summary of the clinical studies, efficacy, and limitations of various common TEF treatment methods in clinical practice^[^ ^16^, ^28^, ^57^, ^106^ ^]^(**Table** **2**).

**Table 2:** The efficacy and the limitations of various common treatment methods for TEF in clinical practice.

Author and Years	Study type	Number of samples	Types of causes	Treatment Method	Efficacy Indicators (Closure Rate)	follow‐up time	limitation
Wang Qingxia et al. (2023) ^[^ ^16^ ^]^	Retrospective cohort study	194	malignant(esophageal cancer or lung cancer)	covered metal stent	94.33%	6 months	3.1% stent displacement
Liu Yao et al. (2023) ^[^ ^28^ ^]^	case series	7	complex recurrent tracheoesophageal fistula	Thoracoscopy plus 3D‐printed bioresorbable airway external	100%	15months	Low number of cases
Gupta et al. (2005) ^[^ ^57^ ^]^	Retrospective cohort study	10	Congenital H‐Type and recurrent tracheoesophageal fistula	Electrocautery and histoacryl glue	90%	3months‐9years	40% patients underwent a second endoscopic surgery
Bibas BJ et al. (2016) ^[^ ^106^ ^]^	Retrospective cohort study	20	Acquired a benign tracheoesophageal fistula	surgical procedures	95%	‐	55% patients complications occurred subcutaneous emphysema and pneumonia

### 8.2 Call for More Clinical Trials and Emerging Technology Research

Despite the progress outlined above, significant gaps persist in the understanding of the long‐term outcomes of various treatment modalities for TEF. Emerging therapies, such as stem cell applications and tissue engineering, have shown preliminary success but require extensive clinical trials to establish their efficacy and safety. Similarly, the long‐term effects of covered metallic stents and electrocoagulation techniques have not been sufficiently studied. It is critical to address these gaps through methodologically rigorous trials involving diverse patient populations to generalize the findings and ensure equitable access to the benefits of advanced therapies.

Technological innovations such as AI and advanced imaging techniques also hold transformative potential in TEF management. For instance, AI‐driven diagnostic tools can enable the early detection and more precise characterization of fistulas and optimize treatment planning. Moreover, the integration of 3D printing technology in presurgical planning has the potential to enhance preparation for anatomical complexities, particularly in recurrent or congenital cases.

Future research should prioritize multidisciplinary collaboration. The integration of thoracic surgery, gastroenterology, radiology, and rehabilitation expertise provides a comprehensive framework for studying new interventions. Furthermore, fostering closer communication among research institutions, regulatory bodies, and funding organizations is essential for accelerating innovation in TEF care. Practical steps include creating dedicated funding streams for TEF research and establishing international TEF registries to track outcomes and identify best practices.

Considering these research imperatives, stakeholders must actively support ongoing studies and foster innovation that aligns with the evolving needs of patients with TEF.

### 8.3 Emphasize the Importance of Multidisciplinary Collaboration and Personalized Treatment Strategies

Treatment of TEF requires a multidisciplinary approach. Successful outcomes hinge on the coordinated efforts of thoracic surgeons, gastroenterologists, nutritional specialists, and rehabilitation clinicians. Collaborative practices such as joint planning of surgical interventions and synchronized postoperative care are critical in reducing complications and enhancing recovery timelines. Specific examples include intraoperative localization techniques for recurrent TEF, which require the combined expertise of thoracic and radiological teams to achieve optimal results.^[^ ^99^ ^]^

Personalized treatment strategies further amplify the effectiveness of TEF management by tailoring interventions to each patient's unique clinical scenario. Factors such as the anatomical complexity of the fistula, underlying etiological mechanisms, and patient preferences should guide the therapeutic choices. Innovative genomics‐based treatment approaches and data‐driven prognostic tools have the potential to further refine these personalized strategies, especially by predicting recurrence risks and tailoring preventive measures for high‐risk individuals.

Advancements in AI and big data analytics promise to revolutionize the personalization of TEF. Future systems could integrate vast patient datasets to develop predictive models for optimal treatment pathways, thereby reducing trial and error in treatment selection. Similarly, biodegradable materials and 3D printing may enable better surgical interventions that are perfectly adapted to individual patient anatomy.

Therefore, a unified and adaptive approach is required to achieve these goals. TEF care must evolve in tandem with emerging technologies to ensure that all stakeholders in the healthcare continuum, from surgeons to rehabilitation specialists, contribute to a holistic treatment paradigm. Personalized and collaborative care supported by technological innovations provides the foundation for achieving durable solutions and improving patient outcomes in this challenging condition.

## Conflict of Interest

The authors declare no conflict of interest.
